# Supplementary material for: Room temperature photochemical synthesis of metal–organic frameworks for enhanced photocatalysis
Source: Nat Commun. 2026 Mar 20;17:4274. doi: 10.1038/s41467-026-70927-w (PMC13168299; doi:10.1038/s41467-026-70927-w)
Supplement: Supplementary file 1 — Supporting Information [file 41467_2026_70927_MOESM1_ESM.pdf]

Supporting Information

Room Temperature Photochemical Synthesis of Metal–Organic Frameworks for Enhanced Photocatalysis

Yong Wang,[1,2,†] Jingzhuo Guan,[3,†] Kush Kumar,[4] Wanting He,[1] Jesus Valdez,[5] Ruiqi Yang,[1] Guoping Hu,[2] Shengyun Huang,[2] Audrey Moores,[5,6,7] Santosh Kumar Meena,[4] Yongfeng Zhou,[3] Yannan Liu,[3]* and Dongling Ma[1]*

[1] Institut National de la Recherche Scientifique, 1650 Boulevard Lionel Boulet, Varennes, Québec J3X 1P7, Canada.

[2] Ganjiang Innovation Academy, Chinese Academy of Sciences, Ganzhou 341119, China.

[3] State Key Laboratory of Synergistic Chem-Bio Synthesis, School of Chemistry and Chemical Engineering, Shanghai Jiao Tong University, Shanghai, China

[4] Department of Chemical Engineering, Indian Institute of Technology Ropar, Rupnagar 140001, Punjab, India.

[5] Facility for Electron Microscopy Research (FEMR), McGill University, Montréal, Canada.

[6] Centre in Green Chemistry and Catalysis, Department of Chemistry, McGill University, Montreal, QC, Canada

[7] Department of Materials Engineering, McGill University, Montreal, QC, Canada

† These authors contributed equally.

* Email: lyannan@sjtu.edu.cn; dongling.ma@inrs.ca

1. Supplementary Method

1.1 Materials

4,4′-bipyridine (⩾99.0%, BPY), N,N-dimethylformamide (⩾99.8%, DMF), polyvinylpyrrolidone (40K MW, PVP), $Co(NO_3)_2·6H_2O$ (⩾98.0%), $Cu(NO_3)_2·3H_2O$ (⩾98.0%), $MnCl_2·H_2O$ (≥97.0%), 2-methylimidazole (99.0%), trimesic acid (94.0%), and methanol (≥99.8%) were purchased from Sigma-Aldrich. Tetrakis(4-carboxyphenyl)porphyrin (>97.0%, TCPP) and ethanol (≥99.5%) were purchased from Fisher Scientific. Triethanolamine (⩾99.0%), sodium sulfate (≥99.0%), chloroplatinic acid (≥37.0%), benzyl alcohol (≥98.0%), and acetonitrile (≥99.8%) were purchased from Sinopharm Chemical Reagent Co., Ltd. All the chemicals were used without further purification.

1.2 Characterization

X-ray diffraction (XRD) tests were carried on a STOE STADI P diffractometer with Cu Kα radiation (1.54178 Å). The morphology and structure of the Co-MOF samples were characterized using a SEM (LYRA3, Tescan) and a TEM (JEOL 2100F). Raman spectroscopy was performed using an inVia Reflex (Renishaw) Raman spectrometer. Thermogravimetric analysis (TGA) was carried on the Netzsch TA thermal analyzer under the flow of Argon gas. UV-vis DRS absorption spectra were recorded on a UV-Vis spectrophotometer equipped with an integrating sphere (Lambda 750, Perkin Elmer). Fourier transform infrared spectroscopy (FTIR) was carried out with a Thermo Scientific Nicolet 6700/Smart iTR spectrometer. X-ray photoelectron spectroscopy (XPS) was acquired using a VG Escalab 220i-XL spectrometer. The LED lamps with different wavelengths (PLS-LED 100C, Perfect Light Ltd), a 300 W Xenon lamp (PLS-SXE300/300UV, Perfect Light Ltd), and a solar simulator equipped with an AM1.5G filter (LCS-100, Newport) were used as the light sources for synthesis. The Co content of the samples was determined by inductively coupled plasma optical emission spectrometry (ICP-OES, PQ9000, Germany). N_2 adsorption–desorption studies were analyzed using a JWGB-BK 300 analyzer at 77 K. The specific surface areas are

calculated by Brunauer-Emmett-Teller (BET) method, and the pore size distributions were determined by the Barrett–Joyner–Halenda (BJH) method using the desorption branch of the isotherms. The ^{1}H NMR spectra were acquired on an AVANCE NEO 400 MHz spectrometer. The samples were dissolved in deuterated DMSO, and the data were collected with 16 scans using a 5 mm NMR tube. Chemical shifts are reported relative to the residual DMSO peak at 2.50 ppm.

1.3 In situ photothermal stability characterization using laser-induced heat within TEM

The samples for in situ TEM characterizations were prepared by drop-casting phoPPF-3 and PPF-3 on ultrathin carbon-supported nickel TEM grids. In situ laser heating experiment was performed using a modified JEM-2100PLUS TEM (JEOL Ltd., Japan) equipped with a femtosecond (fs) pulsed laser (515 nm wavelength, 400 kHz repetition rate, 220 fs pulse width, ~ 90 μm spot size). The TEM was operated in a thermionic mode at 200 keV, enabling in situ excitation, heating, and real-time observation of the samples with nanoscale spatial resolution. The laser fluence was incrementally increased from 0 to 0.45 mJ cm^{-2} while monitoring structural evolution. For each fluence, samples were irradiated for 5 min before image acquisition (0.5 s exposure time). During laser irradiation, the electron beam was blocked to eliminate any possible beam-induced effect.

1.4 Thermogravimetry-differential thermal analysis and mass spectrometry (TG/DTA-MS) measurements

The thermal decomposition of the samples was investigated using a coupled TG/DTA-MS system (STA 449 F5 TGA/DTA Jupiter with QMS 403C Aëolos mass spectrometer). The TG and DTA were connected with MS via a heated capillary tube (T = 300 °C), enabling real-time mass analysis (scan mode, m/z = 1–300). Measurements were performed under flowing Ar (240 mL min^{-1}) using open Al_2O_3 crucibles, with a heating rate of 10 °C min^{-1} and initial sample mass of ~1.46 mg.

1.5 3D electron tomography reconstruction

3D tomography data acquisition was conducted using Talos F200X G2 and the

SerialEM software[1]. For enhanced contrast, STEM dark-field mode was employed for the 3D reconstruction of phoPPF-3. Data were collected over a tilt range of ± 60° with an increment of 2°. The acquired datasets were processed using the IMOD-Etomo software[2], followed by reconstruction via a simultaneous iterations reconstruction technique algorithm (SIRT) with 20 iterations. The visualization and tomogram segmentation were performed using UCSF Chimera software.[3]

1.6 XRD simulation

XRD patterns of the proposed MOFs were simulated using the Reflex module in Materials Studio 2020. Calculations were performed with Cu Kα radiation (λ = 1.5406 Å) over a 2θ range of 3-45° with a step size of 0.02°. Instrumental broadening was modeled using a pseudo-Voigt function with a default half-width of 0.1°. The crystal structures of HKUST-1, ZIF-67, and ZIF-8 were obtained from the CCDC crystal database and subjected to energy minimization using the Forcite module. The detailed cell space groups, lattice constants, and atomic coordinates of phoPPF-3, PPF-3, HKUST-1, ZIF-67, and ZIF-8 are listed in Supplementary Tables 7-11.

1.7 Photoelectrochemical measurements

Photoelectrochemical properties were evaluated using a standard three-electrode system coupled with a CHI760E electrochemical workstation. A saturated Ag/AgCl electrode served as the reference electrode, and a platinum wire was used as the counter electrode.

For photocurrent and electrochemical impedance spectroscopy (EIS) measurements, the working electrode was prepared by applying 100 μL of a slurry onto an ITO glass plate, covering an area of about 1 cm^2. For Mott-Schottky measurements, the working electrode was prepared by applying 100 μL of the slurry onto a glassy carbon electrode (GCE). The slurry was prepared by dispersing 1 mg of the sample in 990 μL of ethanol and 10 μL of Nafion, followed by sonication for 30 min.

Photocurrent measurements were performed in a 0.5 M sodium sulfate (Na_2SO_4) electrolyte under intermittent illumination (20 s light on/off cycles) using a 300 W

Xenon lamp as the light source. EIS measurements were performed with an AC voltage amplitude of 5 mV, a frequency range from 10^5 to 0.1 Hz, and a 0.5 M Na_2SO_4 aqueous solution as the electrolyte. Mott-Schottky curves were generated by applying an AC voltage with frequencies of 500, 1000, and 1500 Hz, over a voltage range of -1 to 1 V in a 0.5 M Na_2SO_4 solution.

1.8 Photocatalytic oxidation of benzyl alcohol

Typically, a mixture of 10 mg phoPPF-3 (or PPF-3), 10 μL benzyl alcohol, 20 μL of 5 wt% chloroplatinic acid, and 10 mg $Co(NO_3)_2$ was prepared in 20 ml acetonitrile within a 40 mL reactor. The mixture was degassed by N_2 and CO_2 for 20 min. CO_2 acts as an oxidant to promote charge separation and as aids the process through regulating reaction pathways. Following 12 h of reaction using a 300 W Xenon lamp as a light source, reactant conversions were determined by GC-MS analysis.

1.9 Photocatalytic H_2 evolution

Typically, 10 mg of phoPPF-3 (or PPF-3) was dispersed in 18 mL of water and 2 mL of triethanolamine within a 40 mL glass vessel. Subsequently, a Pt-loaded photocatalyst was prepared by chemical reduction by adding 20 μL of an 8 wt% aqueous chloroplatinic acid solution. Prior to irradiation, the reaction system was purged with N_2 for 20 min to remove dissolved oxygen. The reaction mixture was subsequently irradiated under a 300 W Xenon lamp for 12 h, with H_2 production quantified by gas chromatography with a TCD detector.

1.10 Models and simulation details

MD simulations were performed to study the self-assembly of Co-MOF in the presence of BPY, PVP, DMF and ethanol under two distinct conditions: (1) constant heating at 353 K and (2) light exposure at 420 nm wavelength.

To investigate the self-assembly of Co-MOF under light exposure, we calculated the temperature increase after the adsorption of 420 nm light.[4] If one or more photons of this wavelength are absorbed by a Co-MOF group initially at thermal equilibrium (288 K) with average vibrational energy $\langle E \rangle$ (excluding zero-point energy), and the excitation energy is redistributed via intramolecular vibrational relaxation across all the

harmonic vibrational modes of the ground electronic state according to Boltzmann statistics, the resulting molecular temperature (*T*) can be determined by satisfying the relation:[5]

$$< \mathrm{E} > + \frac{nhc}{\lambda} = \sum hv_i \, [e^{\frac{hv_i}{k_B T}} - 1]^{-1}$$

Here, the summation extends over all (3N - 6) vibrational modes with frequencies v_i, where n represents the number of absorbed photons, c is the speed of light, h represents Planck's constant, λ is the photon wavelength, and K_B represents the Boltzmann constant. For a Co-MOF system comprising 88 atoms (258 vibrational modes), the calculated temperature rise upon absorption of a single photon (n = 1, λ = 420 nm) is 718 K.

Initially, we optimized the Co-MOF structure under both thermal and light exposure conditions. Atomic charges were determined by fitting the quantum mechanical electrostatic potential at the B3LYP/6-31G* level of theory, while the force-field parameters obtained by the procedure followed in reference,[6] where bonded and van der Waals parameters were taken from the GROMOS parameter set.

Force-field parameters: The GROMOS96 53A6 force field was used for all MD simulations.[7] Detailed configuration parameters of the simulation models are listed in Supplementary Table 12. Force-field parameters for PVP, BPY, DMF and ethanol are generated following the previous reported protocol,[6] with bonded and van der Waals interactions adopted from the GROMOS parameter set.[5] Each simulation produced trajectories of around 100 ns in length. The site-modeled interactions between rigid fragments were represented by a potential combining Lennard-Jones and Coulomb terms.

1.11 DFT calculation

The molecular geometry of TCPP was fully optimized using density functional theory (DFT) with the B3LYP functional and the 6-31G* basis set, as implemented in Gaussian 16. Vibrational frequency analysis confirmed the absence of imaginary frequencies, verifying that the optimized structure corresponded to a true energy minimum.

Subsequently, time-dependent DFT (TD-DFT) calculations were performed on the optimized geometry to investigate ground and excited-state properties. Charge distribution analysis and electron density maps were generated from the optimized structures using Gaussian View for further electronic structure characterization.

1.12 Energy analysis

The energy consumption for photochemical synthesis was briefly estimated. The 420 nm LED illumination lamp has a power of 100 W and operates for 4 h per batch of phoPPF-3, resulting in an estimated energy consumption of 0.4 kWh per batch. For comparison, a typical solvothermal synthesis of PPF-3 requires heating at 80 °C for 24 h. Using a stirring hotplate with a rated power of 1.44 kW (HP88857100, Thermo Scientific), the heater's actual duty cycle under such moderate temperature conditions maybe only 15% ~ 20%. The average hourly power consumption is therefore approximately 1.44 kW × (0.15 ~ 0.20) = 0.22 ~ 0.29 kWh. Over 24 h, this translates to about 5.28 ~ 6.96 kWh per batch.

1.13 The calculation of E-factor and PMI

The environmental factor (E-factor) and process mass intensity (PMI) are crucial metrics in green chemistry used to quantify the waste generated by a chemical process relative to the amount of desired product obtained. They can be calculated by the following formulas.

$$\mathrm{E-factor} = \frac{Total\ mass\ input - Mass\ of\ desired\ product}{Mass\ of\ desired\ product}$$

$$\mathrm{PMI} = \frac{Total\ mass\ input}{Mass\ of\ desired\ product}$$

Total mass input: This is the sum of the mass of everything that enters the reactor and the work-up process, including mass of all precursors and all solvents used. Mass of desired product: The final isolated, dry mass of the MOF.

2. Supplementary Figures

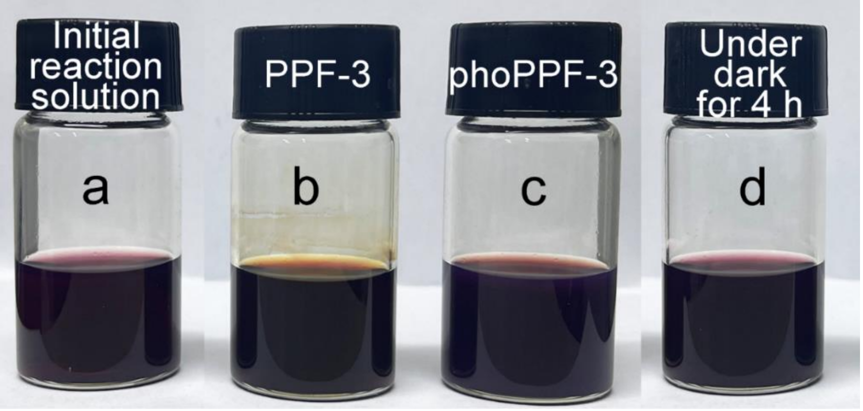

Supplementary Figure 1. Photographs of (a) the initial reaction solution, (b) PPF-3 obtained from solvothermal reaction for 24 h, (c) phoPPF-3 obtained from visible light-driven reaction for 4 h, and (d) solution after reaction under dark for 4 h. Essentially there is no color change in (d) from (a), consistent with no product formation in this case.

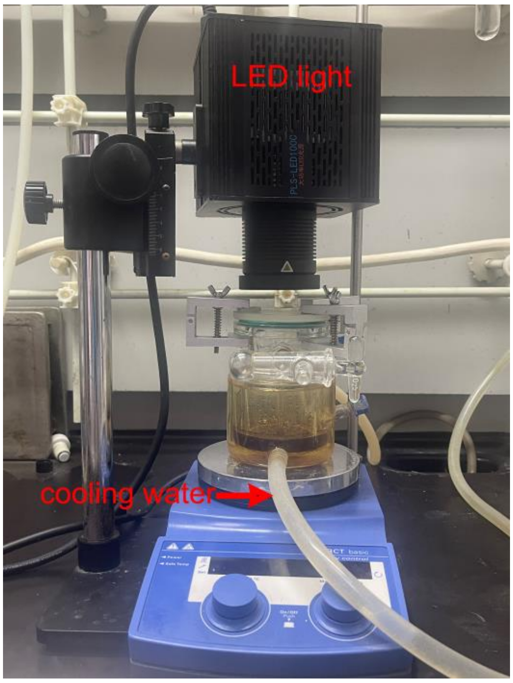

Supplementary Figure 2. Setup of the phoPPF-3 synthesis under light irradiation. The phoPPF-3 sample was synthesized in a sealed reaction bottle and the reaction was carried out at a set temperature by circulating cooling water. The light was placed above the reaction bottle and shined onto the reaction solution through the quartz cover above the reaction bottle. In addition, this reaction was carried out under continuous stirring.

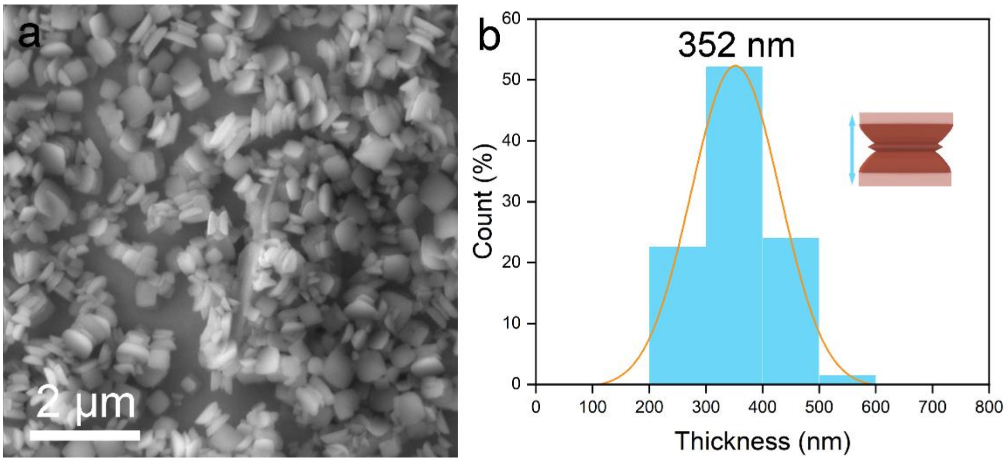

Supplementary Figure 3. (a) SEM image and (b) the corresponding particle thickness distribution histogram of phoPPF-3. The blue arrow in the inset indicates the thickness.

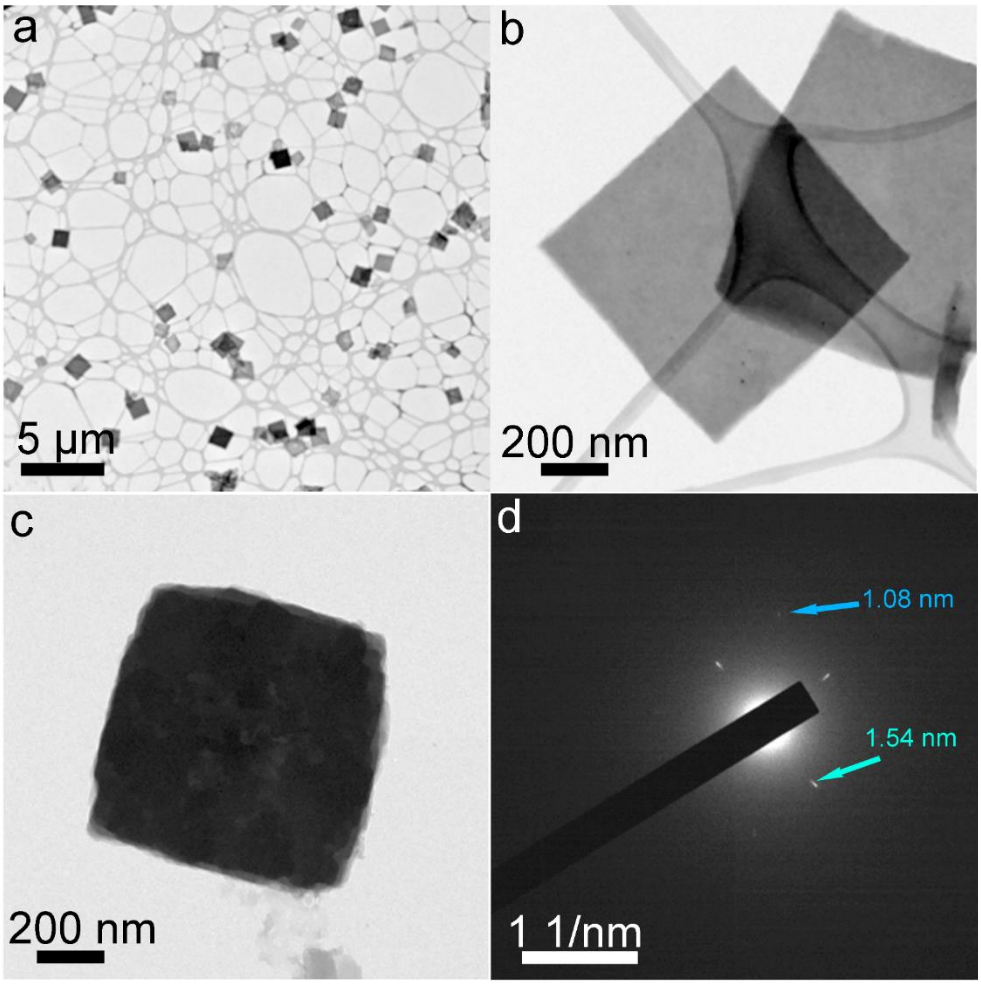

Supplementary Figure 4. (a-c) TEM images and (d) SAED pattern of PPF-3.

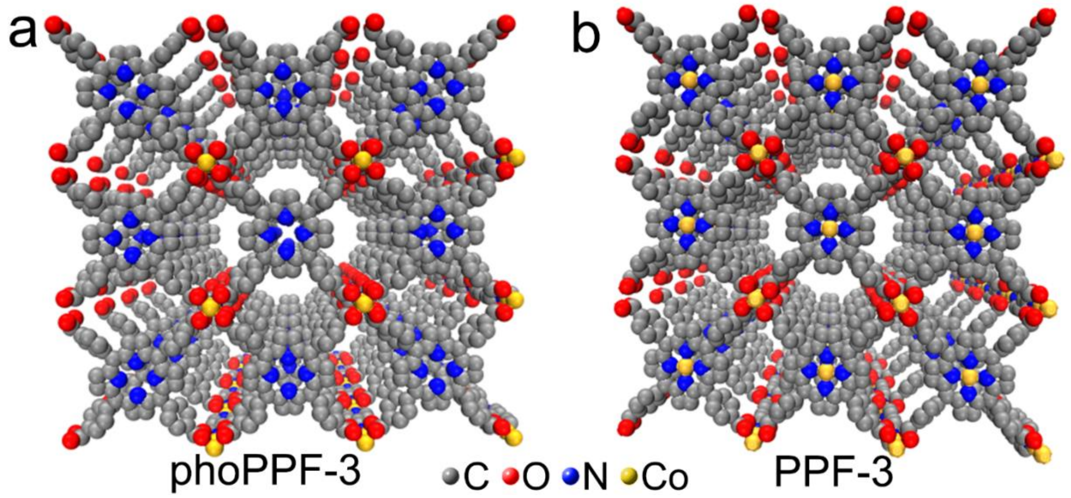

Supplementary Figure 5. Schematic representations of the simulated crystal structures of (a) phoPPF-3 and (b) PPF-3.

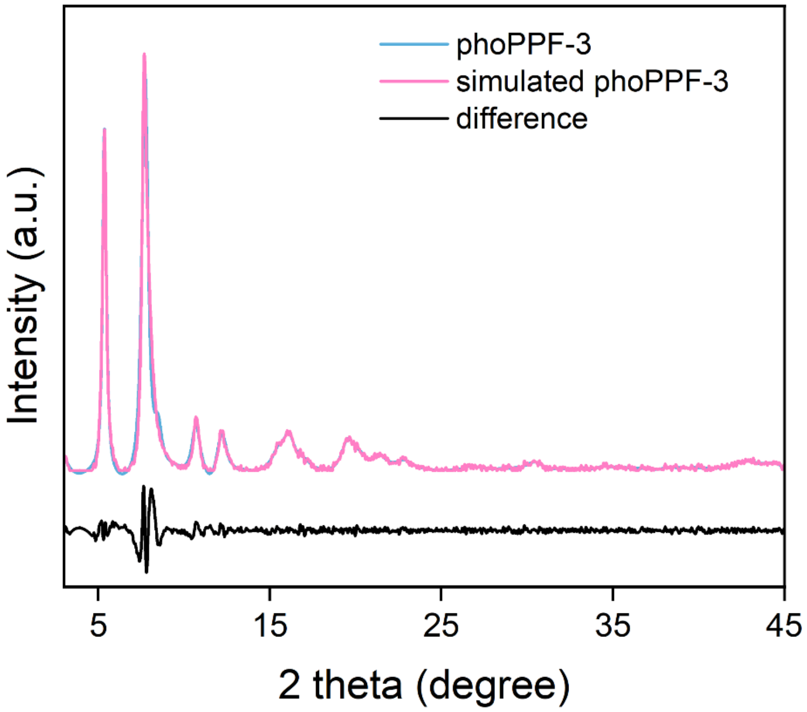

Supplementary Figure 6. Experimental and simulated XRD patterns of phoPPF-3, with a difference plot.

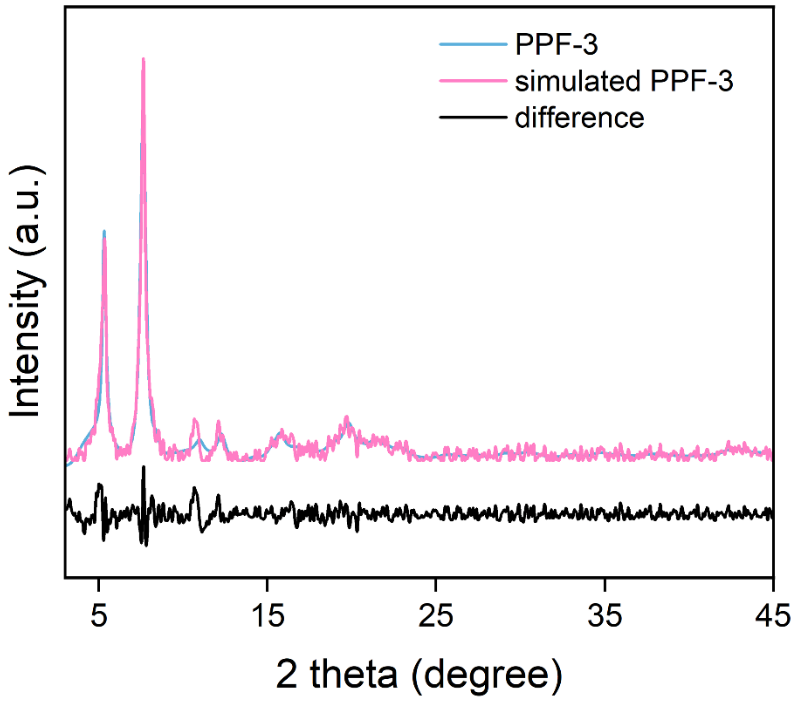

Supplementary Figure 7. Experimental and simulated XRD patterns of PPF-3, with a difference plot.

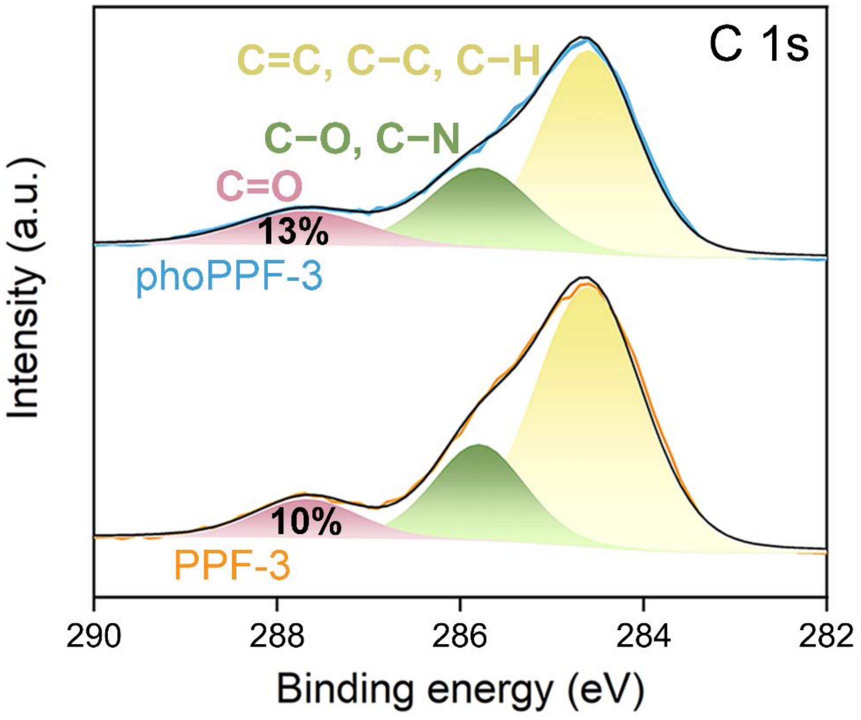

Supplementary Figure 8. XPS spectra of C 1*s* of phoPPF-3 and PPF-3.

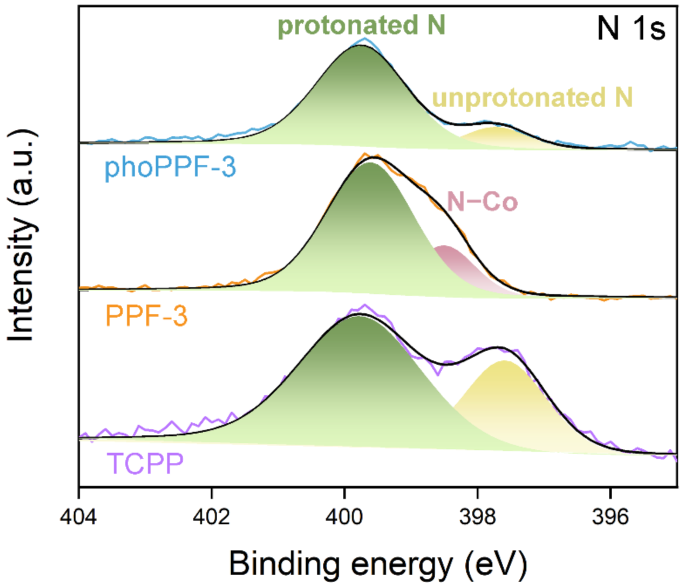

Supplementary Figure 9. XPS spectra of N 1*s* of phoPPF-3, PPF-3, and TCPP. The XPS N 1*s* spectrum of TCPP exhibits two types of nitrogen: a lower binding energy peak for the unprotonated nitrogen (–C=N–) and a higher energy peak of the protonated nitrogen (–NH–).[8,9]

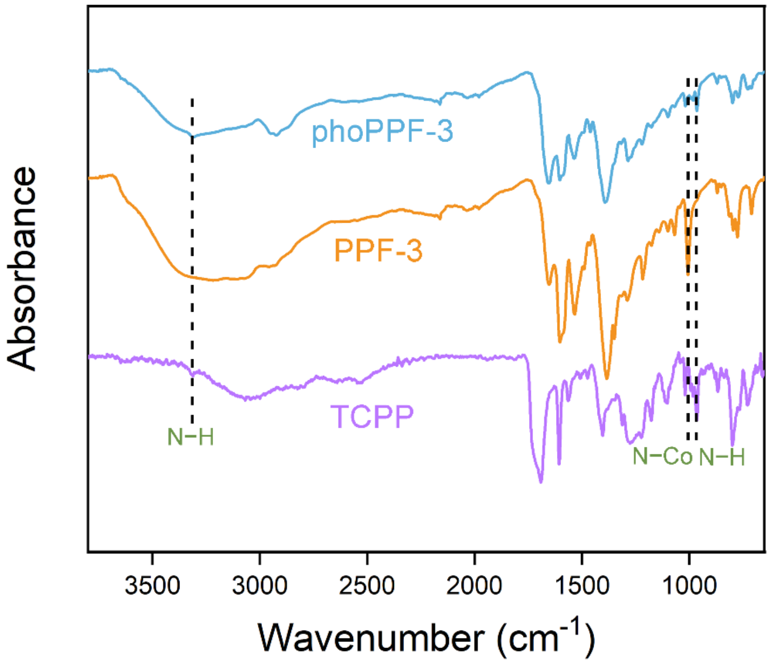

Supplementary Figure 10. FTIR spectra of phoPPF-3, PPF-3, and TCPP.

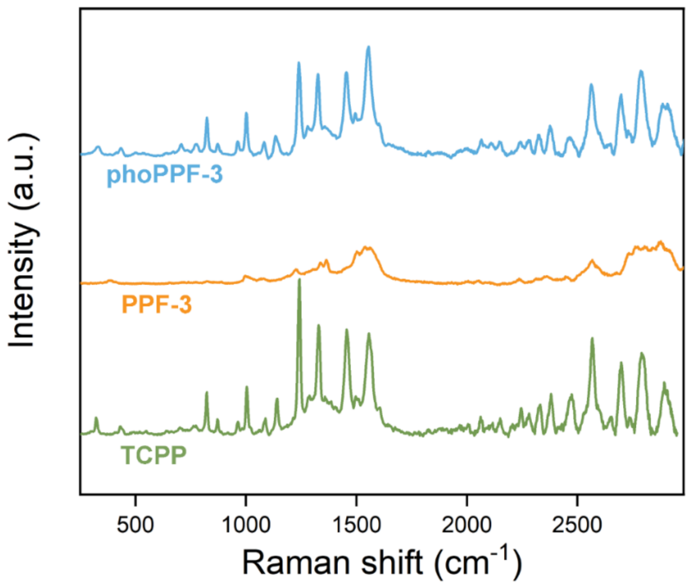

Supplementary Figure 11. Raman spectra of TCPP, phoPPF-3, and PPF-3.

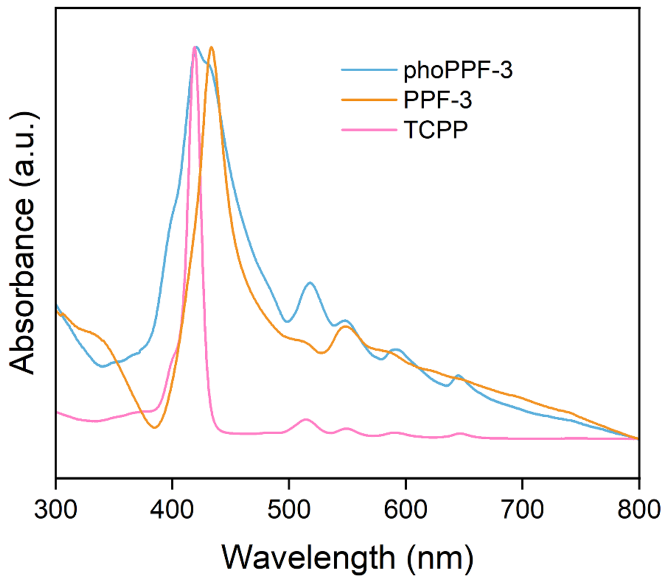

Supplementary Figure 12. UV-vis spectra of phoPPF-3, PPF-3, and TCPP in DMF/ethanol (3:1, v/v) solution. TCPP exhibited characteristic Q bands at 515, 550, 591, and 646 nm. PPF-3 showed a merged Q band at 548 nm, consistent with its UV-vis DRS result, suggesting the retention of Co-porphyrin coordination during dispersed solution. However, phoPPF-3 in solution showed Q bands at 517, 548, 591, 645 nm, suggesting a lack of central coordination of the TCPP linkers.

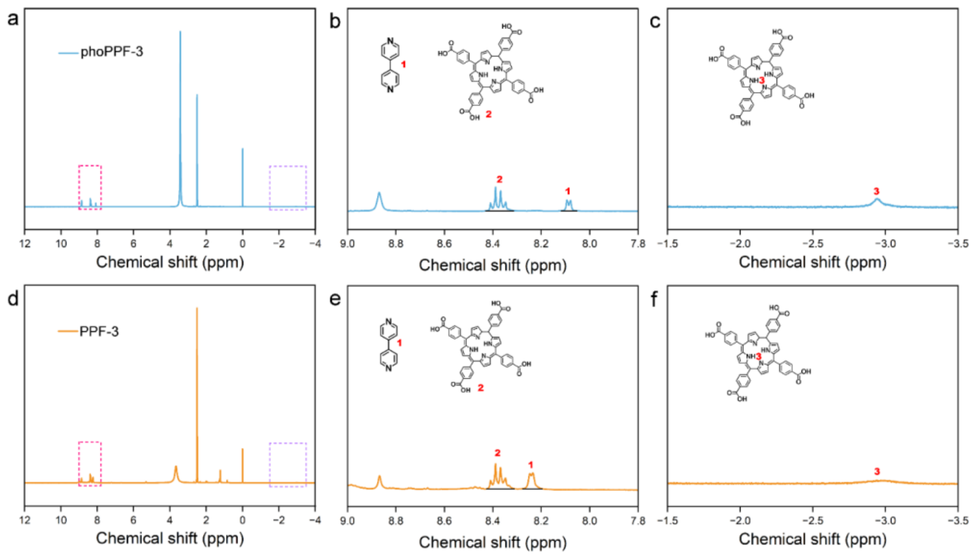

Supplementary Figure 13. The ^{1}H NMR spectra of the digested solutions of (a and b) phoPPF-3 and (c and d) PPF-3. (b and c) and (e and f) show magnified views of the areas indicated in (a) and (d), respectively. For phoPPF-3, the presence of a small peak, which signifies H within the porphyrin ring post acid dissociation, demonstrates the absence of intra-ring Co ion coordination.

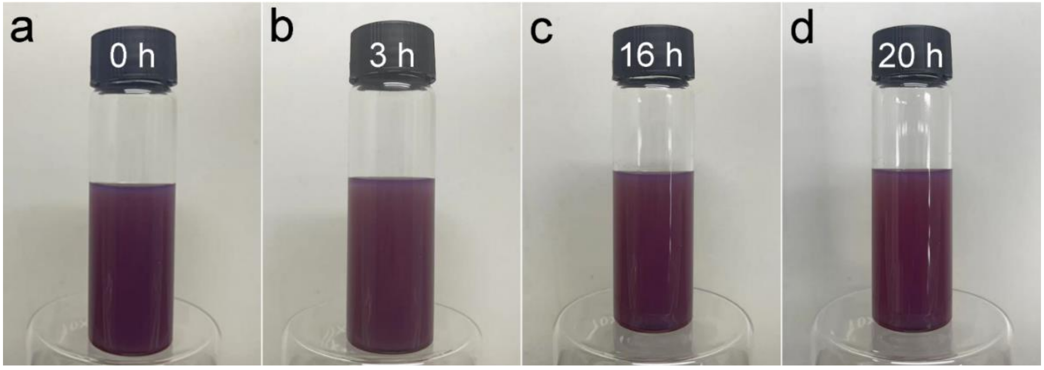

Supplementary Figure 14. Dispersion stability of the phoPPF-3 suspension under static conditions. Photographs of phoPPF-3 dispersed in a DMF/ethanol (3:1) solvent at (a) 0 h, (b) 3 h, (c) 16h, and (d) 20 h.

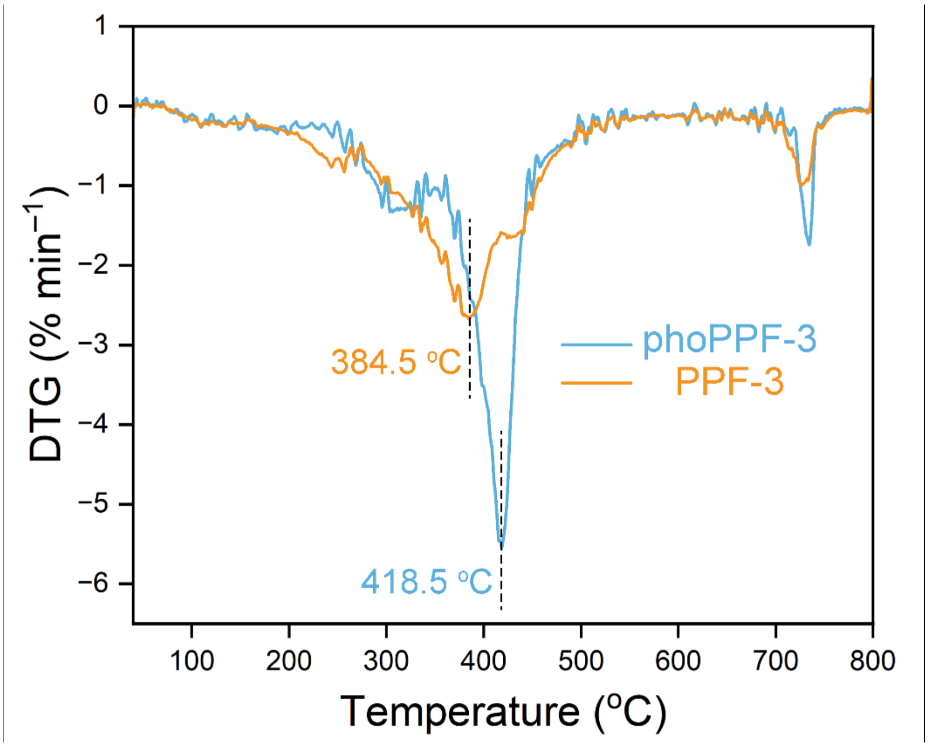

Supplementary Figure 15. DTG curves of phoPPF-3 and PPF-3 obtained under an Argon atmosphere.

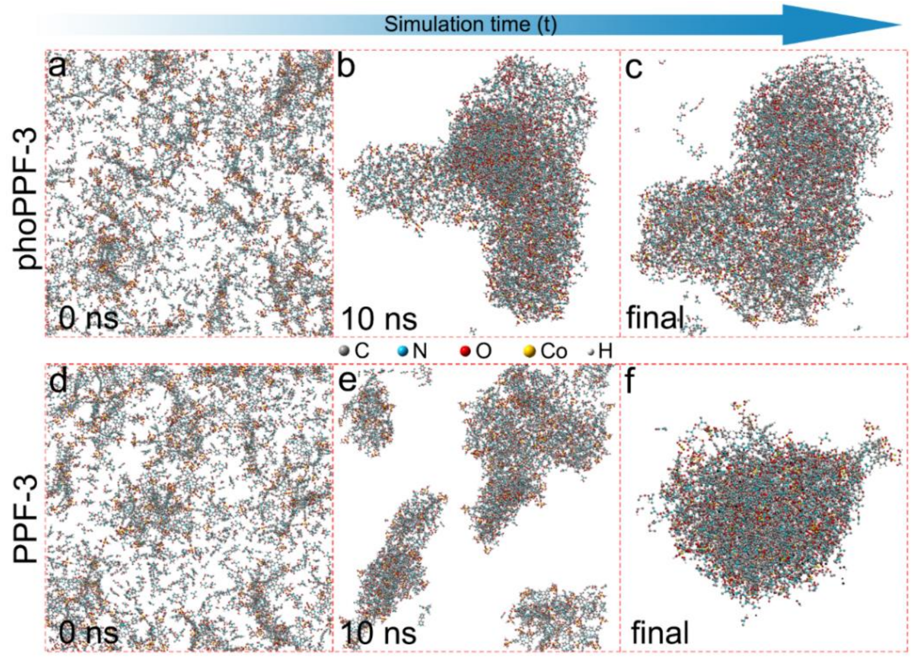

Supplementary Figure 16. Snapshots of the molecular dynamics simulation at a selected time for (a-c) phoPPF-3 and (d-f) PPF-3.

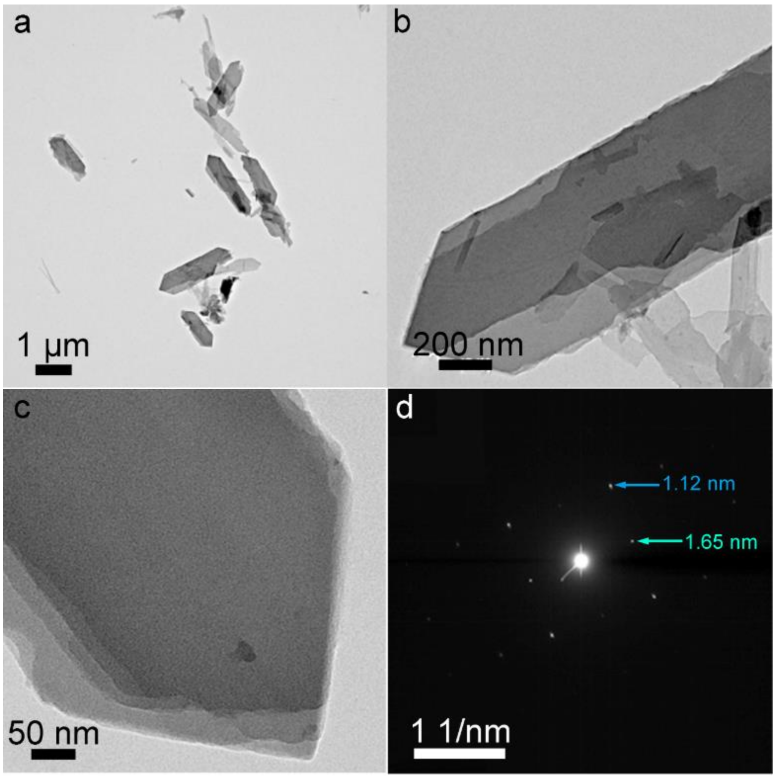

Supplementary Figure 17. (a-c) TEM images and (d) FTT pattern of phoPPF-3$_{(\text{no PVP})}$ prepared without the addition of PVP. The lattice spacings of 1.12 and 1.65 nm are larger than those observed for phoPPF-3, indicating weaker interlayer interactions.

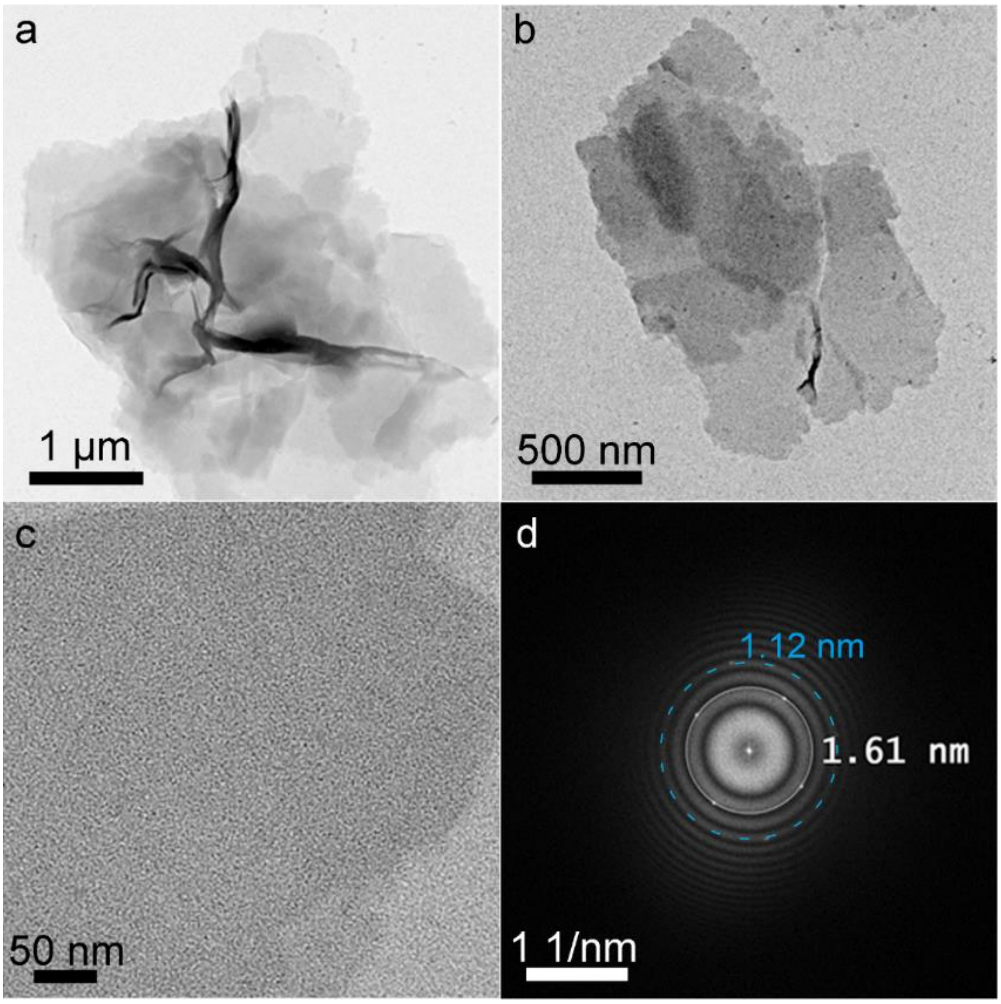

Supplementary Figure 18. (a-c) TEM images and (d) FTT pattern of phoPPF-3 prepared without the addition of BPY. The lattice spacings of 1.12 and 1.61 nm are larger than those observed for phoPPF-3, which may be caused by weaker interlayer interactions.

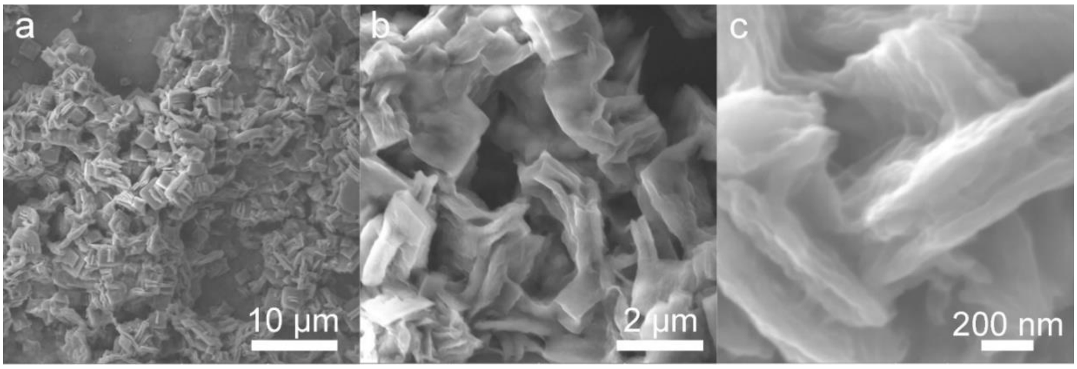

Supplementary Figure 19. (a-c) SEM images of phoPPF-3 prepared without the addition of BPY and PVP.

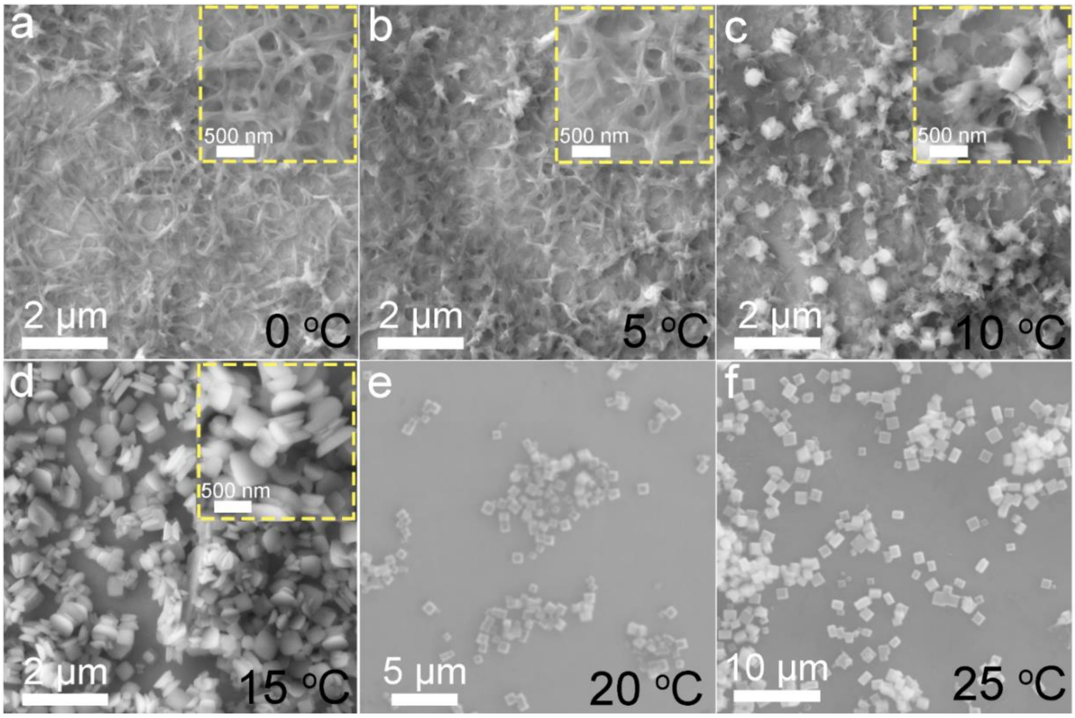

Supplementary Figure 20. SEM images of phoPPF-3 synthesized at temperatures of (a) 0 °C, (b) 5 °C, (c) 10 °C, (d) 15 °C, (e) 20 °C, and (f) 25 °C. The insets in (a)-(d) present zoomed-in SEM images of the selected areas.

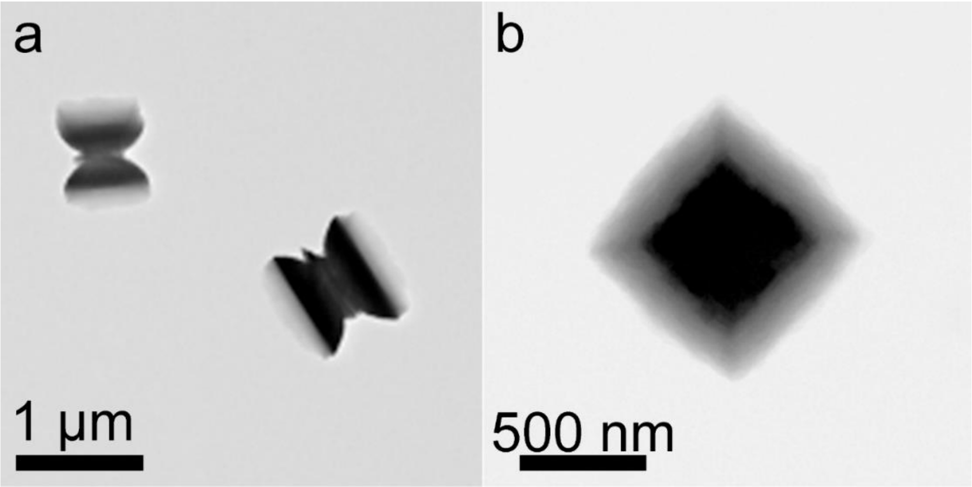

Supplementary Figure 21. (a and b) TEM images of phoPPF-3 prepared under 365 nm LED light.

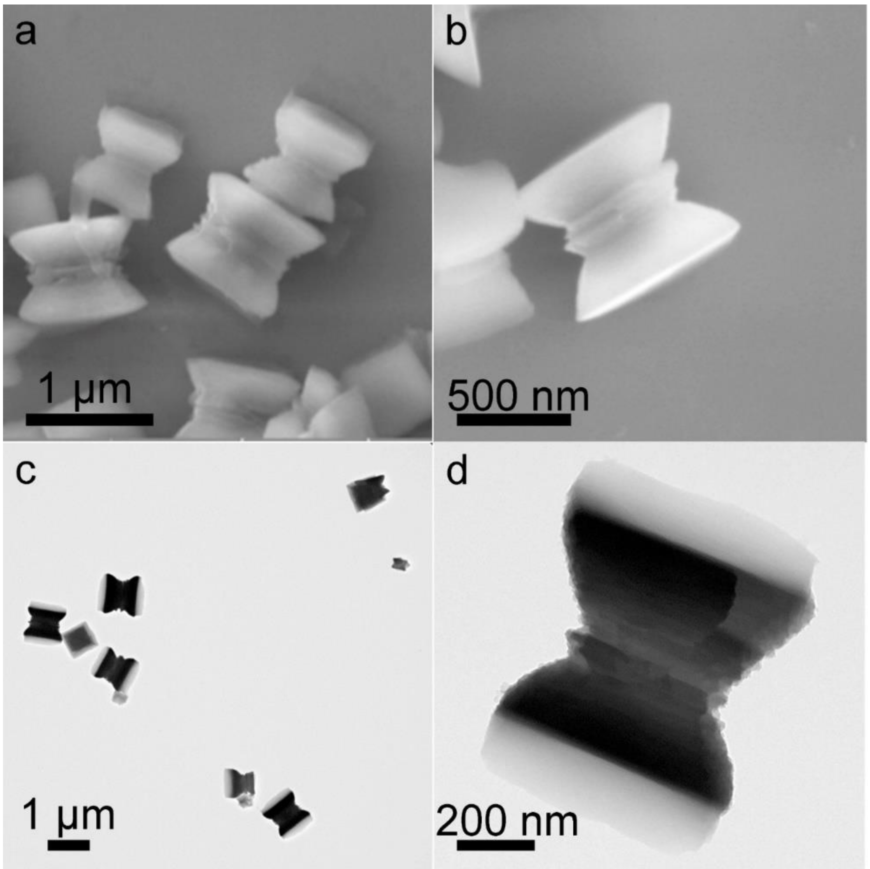

Supplementary Figure 22. (a and b) SEM and (c and d) TEM images of phoPPF-3 prepared under 495 nm LED light.

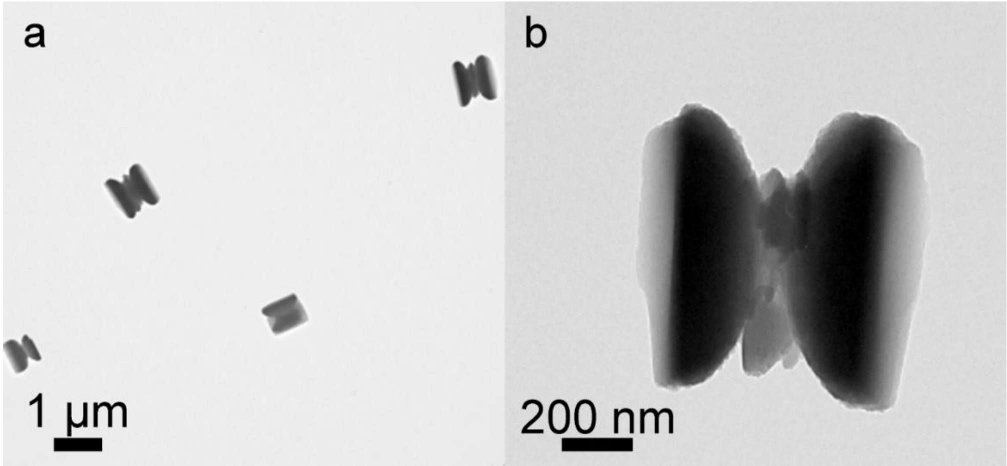

Supplementary Figure 23. (a and b) TEM images of phoPPF-3 prepared under 595 nm LED light.

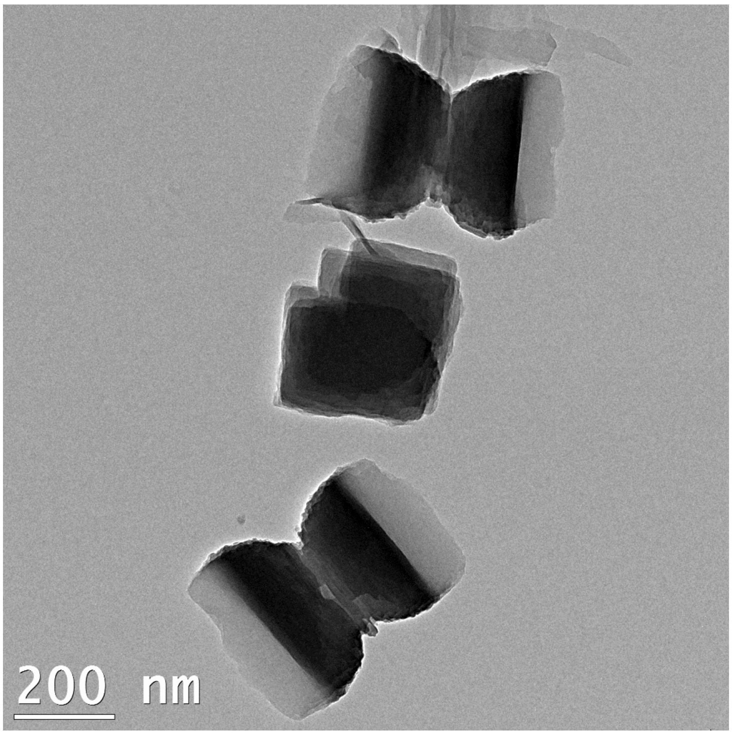

Supplementary Figure 24. TEM image of phoPPF-3 prepared under 300 W Xenon light.

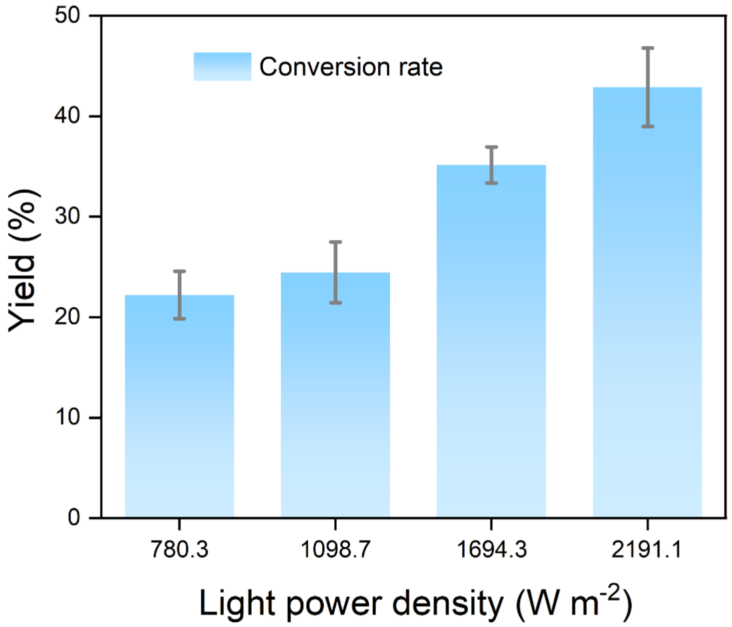

Supplementary Figure 25. The yield of phoPPF-3 varies with the power density of Xenon lamp. The error bars indicate one standard deviation based on three measurements.

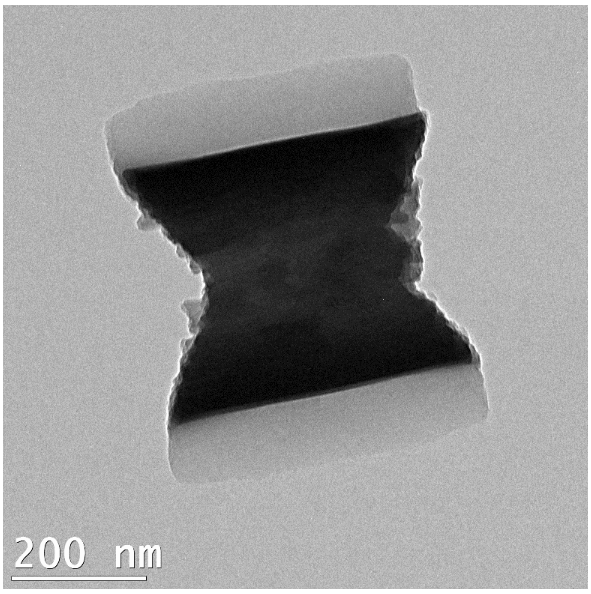

Supplementary Figure 26. SEM image of phoPPF-3 prepared under one sun light (1000 W m^{-2}).

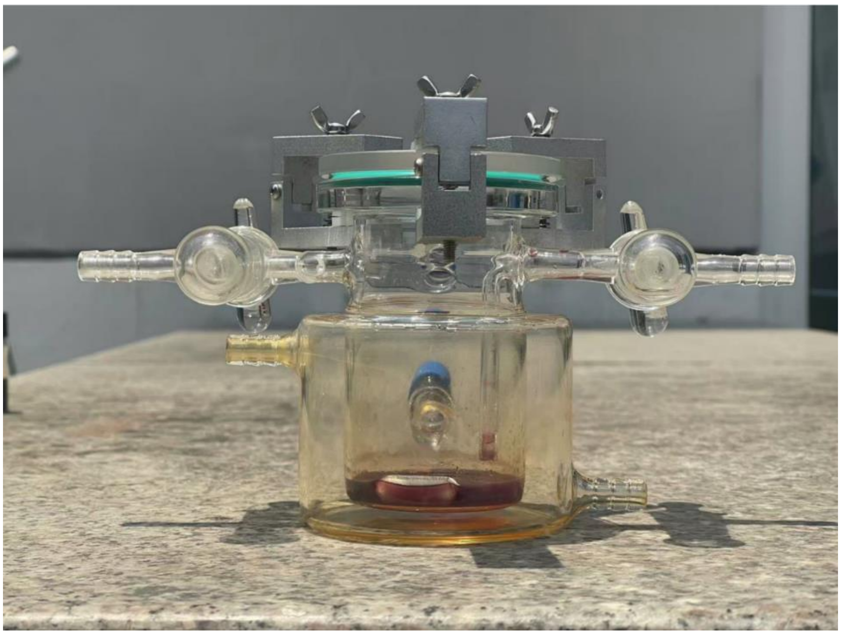

Supplementary Figure 27. Direct light-driven synthesis of phoPPF-3 enabled by natural sunlight. The testing was performed on May 30, 2025 (11:40 AM–15:40 PM, local time) under ambient conditions in Shanghai, China.

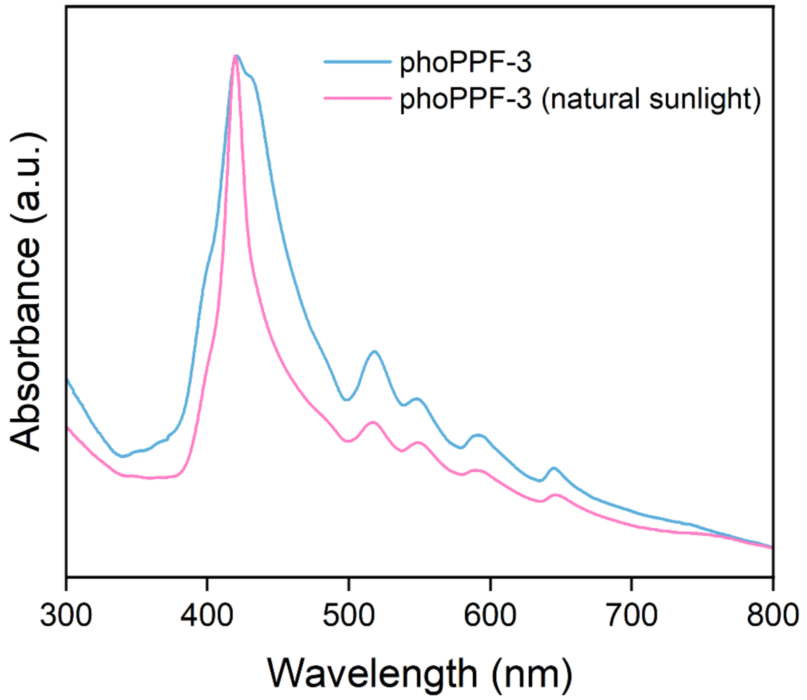

Supplementary Figure 28. UV-vis spectra of phoPPF-3 and phoPPF-3 (natural sunlight) obtained under the sun.

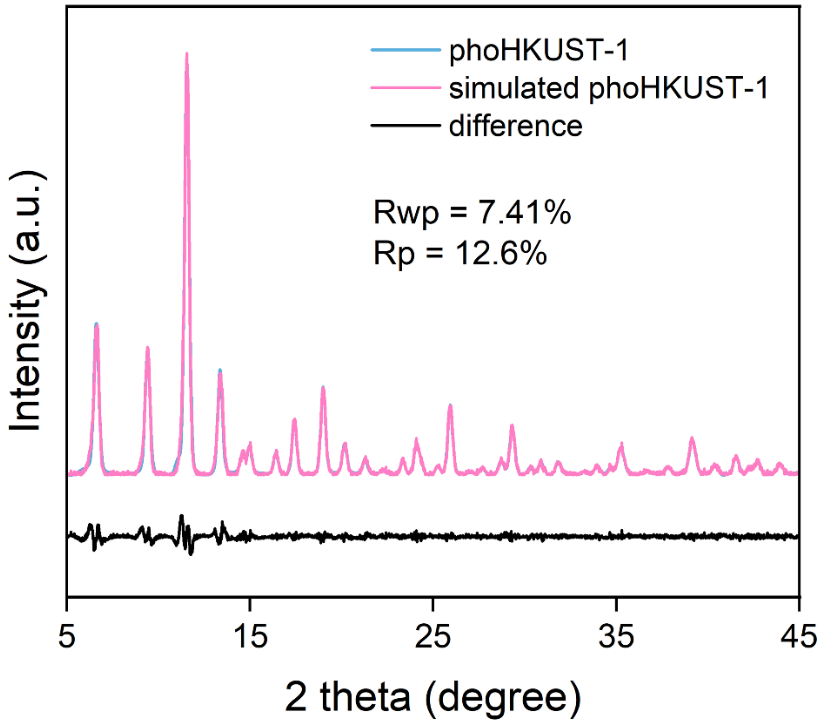

Supplementary Figure 29. Experimental and simulated XRD patterns of phoHKUST-1, with a difference plot.

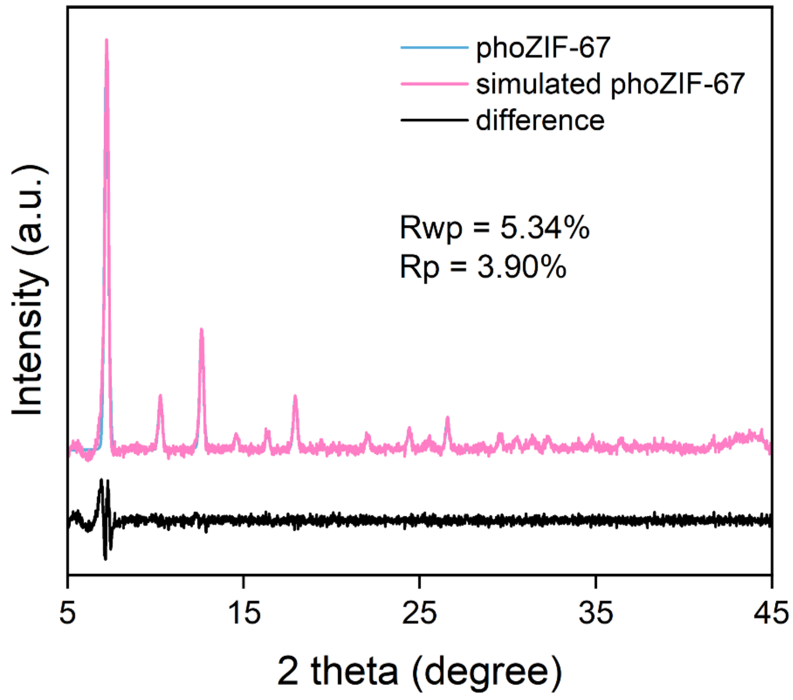

Supplementary Figure 30. Experimental and simulated XRD patterns of phoZIF-67, with a difference plot.

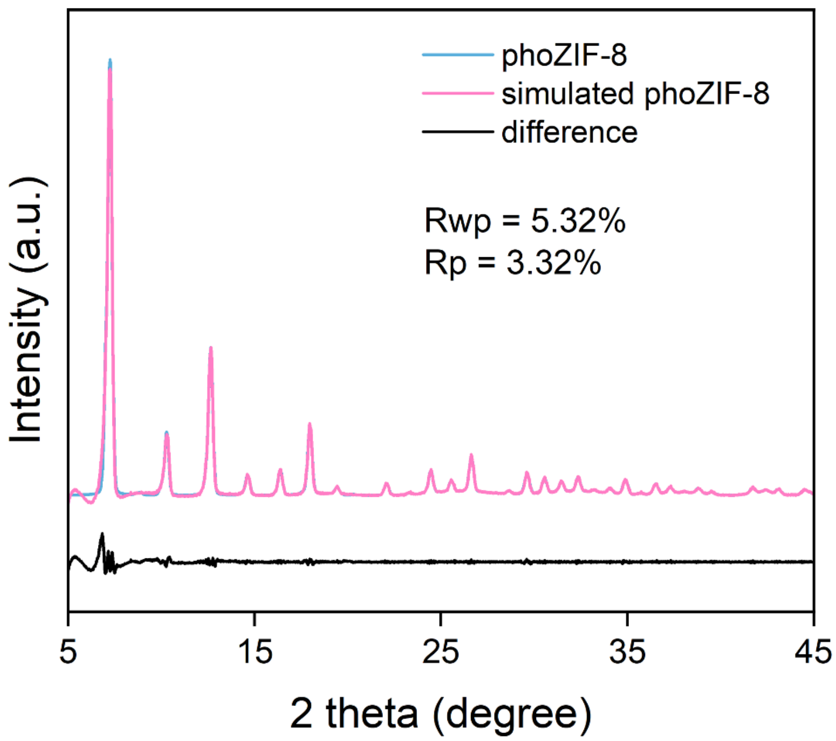

Supplementary Figure 31. Experimental and simulated XRD patterns of phoZIF-8, with a difference plot.

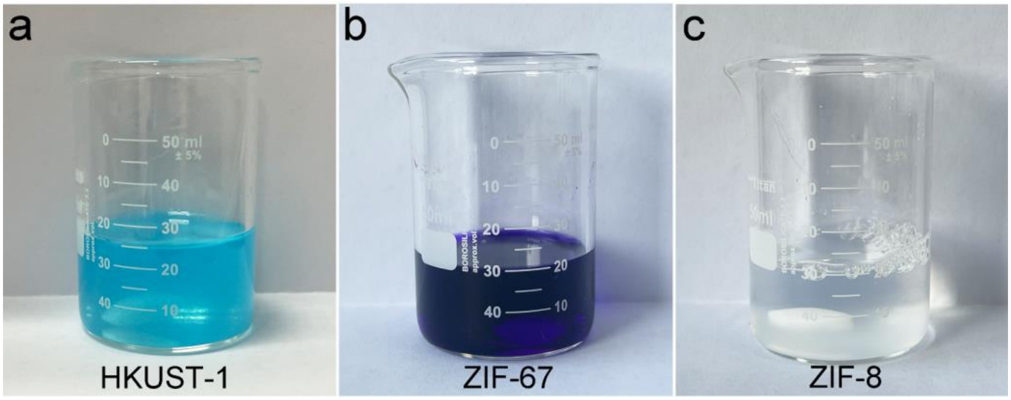

Supplementary Figure 32. Photographs of the reaction mixtures for (a) HKUST-1, (b) ZIF-67, and (c) ZIF-8.

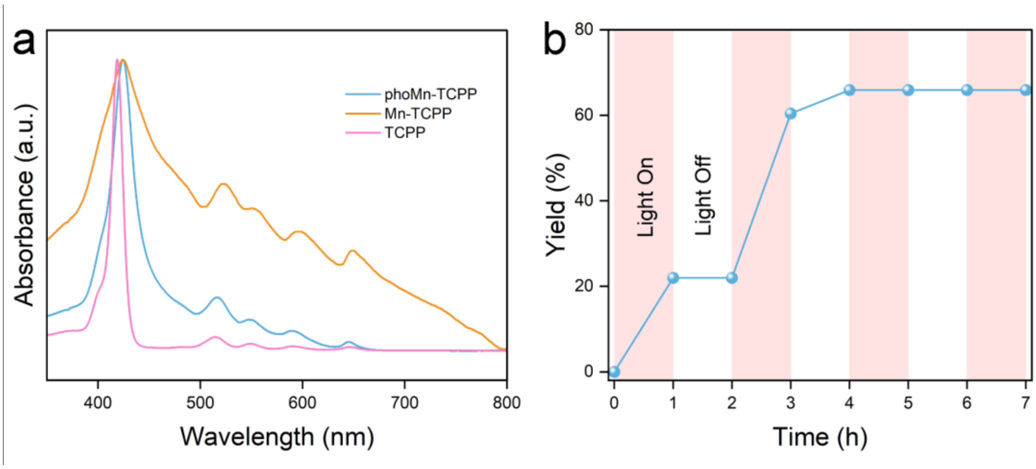

Supplementary Figure 33. (a) UV-vis spectra of TCPP, Mn-TCPP, and phoMn-TCPP. (b) Time-dependent yield variation of phoMn-TCPP under light on and light off conditions. Test conditions: the reaction substrate was three times the standard amount, and 2 mL of reaction solution was taken every 1 h for yield determination. Meanwhile, the light source was cycled between 1 h of light on and 1 h of light off.

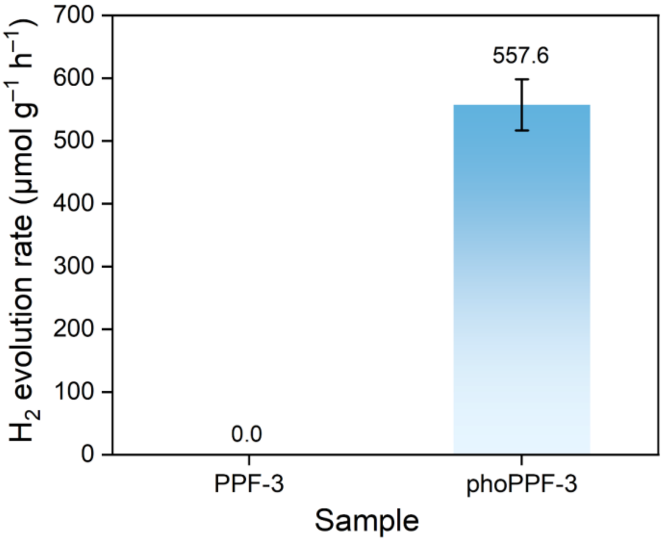

Supplementary Figure 34. Photocatalytic H_2 evolution activities of PPF-3 and phoPPF-3 over 12 h under the following photocatalytic conditions: 25 °C, 10 mg of photocatalyst, and a solution of 18 mL water with 2 mL triethanolamine. The error bars indicate one standard deviation based on three measurements.

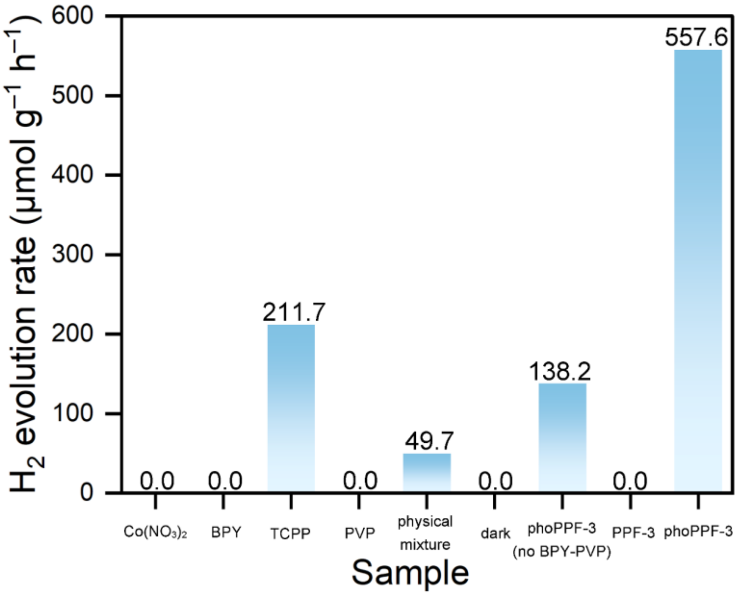

Supplementary Figure 35. Photocatalytic H_2 evolution activities of $Co(NO_3)_2$, BPY, TCPP, PVP, physical mixture (physical mixing of precursors for the synthesis of phoPPF-3), dark (phoPPF-3), phoPPF-3 (no BPY-PVP), PPF-3, and phoPPF-3 over 12 h under the following photocatalytic conditions: 25 °C, 10 mg of photocatalyst, and a solution of 18 mL water with 2 mL triethanolamine. Among the isolated precursors of

$Co(NO_3)_2$, BPY, TCPP, and PVP, only TCPP exhibited hydrogen production activity (211.7 μmol g^{-1} h^{-1}), attributed to its inherent photoactivity. The physical mixture of the components and PPF-3 showed significantly lower activities of 49.7 and 0.0 μmol g^{-1} h^{-1}, respectively. These reductions may be due to the uncoordinated Co^{2+} and Co^{2+} coordinated at the porphyrin center acting as an electron scavenger, hindering efficient hydrogen evolution. In contrast, the phoPPF-3 (no BPY-PVP) and phoPPF-3 exhibited hydrogen production activities of 138.2 and 557.6 μmol g^{-1} h^{-1}, respectively. These comparative results strongly indicate that the specific crystal structure and the controlled incorporation of components within of phoPPF-3 are critical factors for its superior catalytic performance, facilitating efficient charge separation and utilization for hydrogen generation.

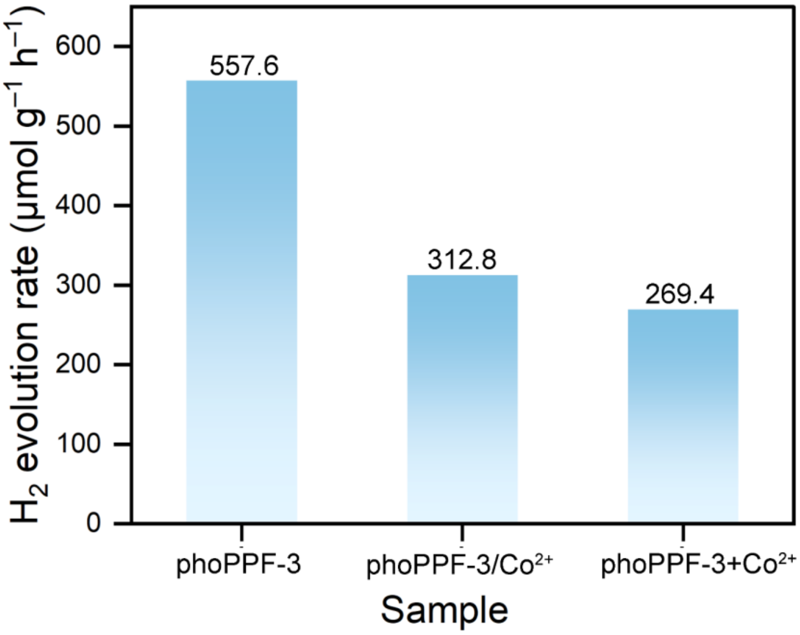

Supplementary Figure 36. Photocatalytic H_2 evolution activities of phoPPF-3, phoPPF-3/Co^{2+}, and phoPPF-3+Co^{2+} under the following photocatalytic conditions: 25 °C, 10 mg of photocatalyst, and a solution of 18 mL water with 2 mL triethanolamine. The phoPPF-3/Co^{2+} sample was synthesized by heating a solution of phoPPF-3 and $Co(NO_3)_2$ at 80 °C for 12 h, while the phoPPF-3+Co^{2+} sample was obtained by simple physical mixing of the two components in solution. The results show that phoPPF-

3/Co^{2+} exhibited negligible hydrogen production activity (312.8 μmol g^{-1} h^{-1}). This value is slightly higher than that of phoPPF-3+Co^{2+} (269.4 μmol g^{-1} h^{-1}) but significantly lower than that of phoPPF-3 (557.6 μmol g^{-1} h^{-1}). These findings strongly suggest that the crystal structure of phoPPF-3 are indeed critical factors for its superior catalytic performance, rather than simply the presence of Co centers within a porphyrin framework. Furthermore, the presence of Co^{2+} demonstrated a negative effect on the photocatalytic performance for hydrogen production, which is consistent with the poor photocatalytic performance observed for PPF-3 (0.0 μmol g^{-1} h^{-1}). The underlying reason may be that uncoordinated Co^{2+} and Co^{2+} coordinated at the porphyrin center act as an electron scavenger, capturing photogenerated electrons and impeding the hydrogen generation process.

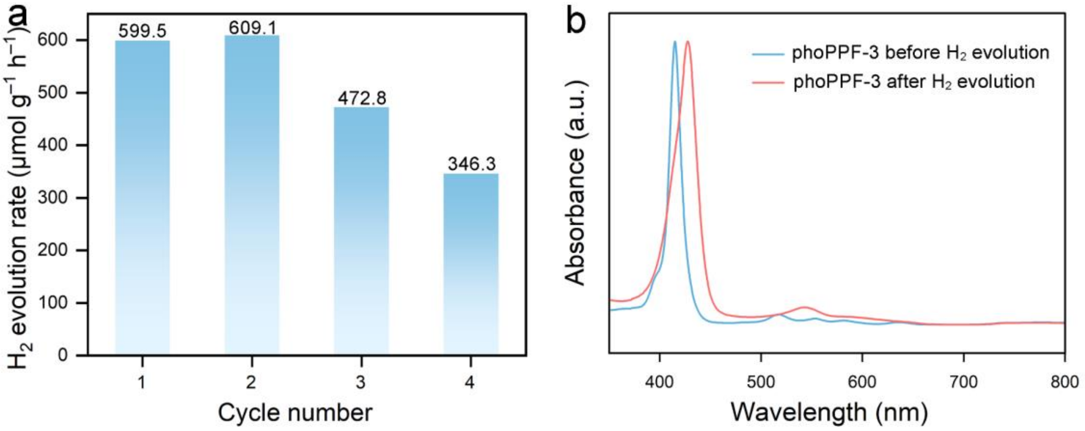

Supplementary Figure 37. (a) Cyclic stability testing of phoPPF-3 for photocatalytic H_2 production. The photocatalytic conditions for each cycle (0.5 h) were maintained at 25 °C with 10 mg of phoPPF-3 in a solution of 18 mL water and 2 mL triethanolamine. (b) UV-Vis spectra of phoPPF-3 in the reaction suspensions before and after four cycles of photocatalytic H_2 production. The phoPPF-3 exhibited a degree of reusability over short-term cycles. However, its long-term photocatalytic stability remains a significant challenge. Notably, the catalytic performance shows a marked decline by the fourth cycle. To investigate this degradation, we compared the UV-Vis spectra of phoPPF-3 before and after the photocatalytic reaction. For the post-reaction sample, the observed red shift in the Soret band and the merging of the Q bands indicate a structural

transformation. These results indicate that after photocatalytic reaction, Co^{2+} ions may have coordinated into the porphyrin center, leading to the observed reduction in photocatalytic performance.

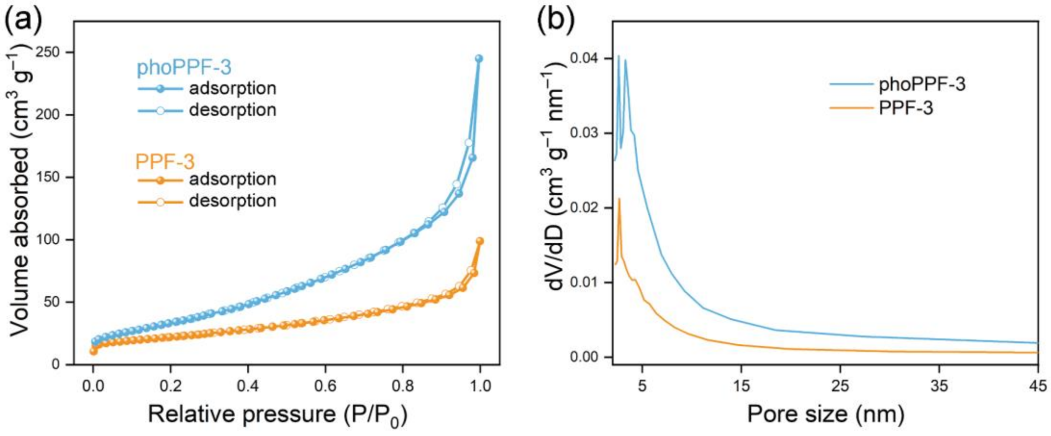

Supplementary Figure 38. (a) Nitrogen adsorption–desorption isotherms and (b) the corresponding BJH pore size distributions of phoPPF-3 and PPF-3.

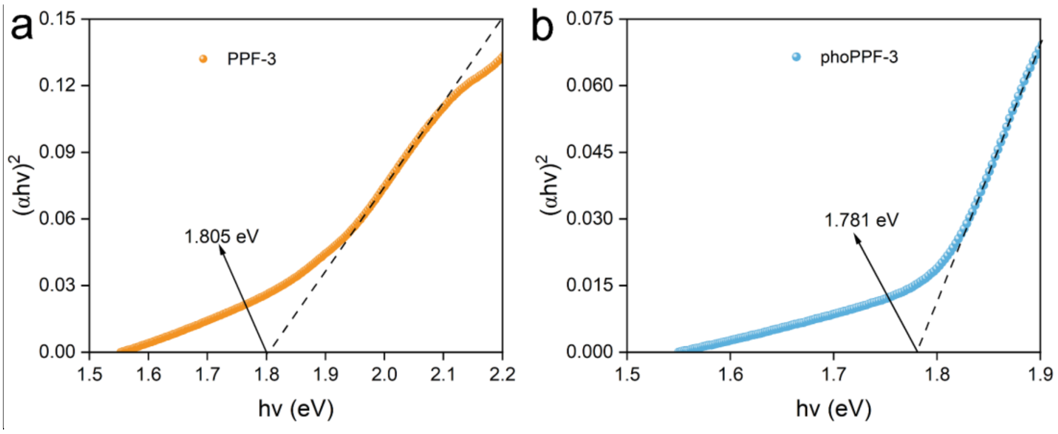

Supplementary Figure 39. Tauc plots of (a) PPF-3 and (b) phoPPF-3.

3. Supplementary Tables

Supplementary Table 1. The ratio of C species calculated by the XPS peak area.

Ratio (%)	C=C, C−C, C−H	C−O, C−N	C=O
PPF-3	68	22	10
phoPPF-3	61	26	13

Supplementary Table 2. The coordination number (CN) of the MOF during light and solvothermal conditions at corresponding RDF peaks.

CN	Conditions	RDF peaks					
		1st	2nd	3rd	4th	5th	6th
MOF	Light	1.612	2.071	2.305	5.617	13.166	17.231
	Solvothermal	1.629	2.185	2.345	5.632	14.166	17.763
BPY	Light	0	0	0	0.026	0.571	1.153
	Solvothermal	0	0	0	0.021	0.623	1.243
PVP	Light	0	0	0.004	0.004	0.004	0.018
	Solvothermal	0	0	0	0	0.008	0.015
DMF	Light	0	0	0.003	0.021	0.852	1.790
	Solvothermal	0	0	0	0.012	0.866	1.701
Ethanol	Light	0	0.003	0.003	0.003	0.143	0.280
	Solvothermal	0	0	0	0.004	0.121	0.387

Supplementary Table 3. Detailed electronic structure parameters for TCPP and TCPP* obtained from DFT simulations.

	TCPP	TCPP*
Dipole moment	2.279 D	2.039 D
Polarizability	766.78 a.u.	848.02 a.u.
N1 charge distribution	-0.773	-0.771
N2 charge distribution	-0.774	-0.776

N3 charge distribution	-0.688	-0.667
N4 charge distribution	-0.690	-0.672

Supplementary Table 4. Performance comparison of PPF-3 and phoPPF-3 in the photocatalytic benzyl alcohol oxidation reaction. The turnover number (TON) and turnover frequency (TOF) were calculated based on the Co content.

Sample	Benzaldehyde yield (%)			Mean value (%)	Standard deviation	TON	TOF (h^{-1})
	1	2	3				
PPF-3	9.18	11.25	10.01	~10.15	1.04	49.83	4.15
phoPPF-3	13.95	14.4	12.13	~13.49	1.20	76.70	6.39

Supplementary Table 5. BET surface areas, average pore sizes, and total pore volumes of phoPPF-3 and PPF-3.

Sample	BET surface area (m^2 g^{-1})	Average pore size (nm)	Total pore volume (m^3 g^{-1})
phoPPF-3	127.99	10.29	0.33
PPF-3	76.85	6.71	0.13

Supplementary Table 6. Energy consumption and throughput for the photochemical synthesis.

**Method**	**Energy source**	**Reaction time (h)**	**Temperature (℃)**	**Energy consumption (kWh/mg product)**	**Product throughput (mg/batch)**	**Potential Throughput (mg/day)**
Photochemical Synthesis	420 nm LED lamp	4	15	0.2	2.00	12.00 (6 batches)
Photochemical Synthesis	Sunlight	4	~30	0	0.73	1.46 (2 batches)
Solvothermal Synthesis	Hotplate	24	80	1.32~1.74	4.00	4.00 (1 batch)

Supplementary Table 7. Cell space groups, lattice constants, and atomic coordinates of phoPPF-3.

Atoms	P1, a = 16.1054 Å, b = 16.6855 Å, c = 13.1579 Å, $\alpha = \beta = \gamma = 90°$		
	x	y	z
C	1.03357	1.32824	0.00262
N	1.10175	1.28122	0.01317
C	1.16697	1.32921	0.00327
C	1.13809	1.41001	-0.02692
C	1.05689	1.40936	-0.02344
C	0.95223	1.30496	0.02121
C	0.92976	1.22552	0.03911
N	0.97891	1.16073	0.02577
C	0.93064	1.09679	0.04282
C	0.85227	1.12118	0.06883
C	0.85177	1.20277	0.06665
C	0.95407	1.01605	0.03002
C	1.03636	0.99142	0.017
N	1.10378	1.04044	0.02094
C	1.17016	0.99168	0.01105
C	1.14287	0.90776	0.00089
C	1.0615	0.90774	0.00342
C	1.25407	1.01655	0.01613
C	1.27943	1.09785	0.02146
N	1.2279	1.16179	0.01329
C	1.27821	1.22732	0.01754
C	1.36089	1.20492	0.02922
C	1.36173	1.12304	0.03074
C	1.25124	1.30724	0.00715
C	0.88739	0.95439	0.02735
C	1.31902	0.95326	0.01582
C	0.88671	1.36723	0.02121
C	1.31396	1.3728	0.00752
C	0.82609	0.95182	-0.05327

C	0.75906	0.89881	-0.05275
C	0.75257	0.84684	0.02771
C	0.81475	0.84865	0.10799
C	0.88147	0.90244	0.10794
C	1.33119	0.907	-0.07327
C	1.39185	0.84709	-0.07339
C	1.44222	0.83321	0.01547
C	1.43028	0.88019	0.10416
C	1.36856	0.939	0.10474
C	1.31872	1.43128	0.08719
C	1.37331	1.49625	0.0849
C	1.42503	1.50346	0.00375
C	1.42069	1.44431	-0.0758
C	1.36523	1.37979	-0.07404
C	0.88831	1.42743	0.0985
C	0.8257	1.48516	0.09961
C	0.76015	1.48306	0.02353
C	0.75859	1.42237	-0.05327
C	0.82165	1.36513	-0.05484
C	0.6916	1.54414	0.02141
C	1.48454	1.5736	0.00595
C	0.67994	0.78973	0.02361
C	1.51233	0.77331	0.01637
O	0.6994	1.59759	0.10551
O	0.69248	1.58611	-0.06796
O	1.52976	1.57297	-0.08126
O	1.53849	1.57014	0.09283
O	1.51082	0.7238	0.09943
O	1.5157	0.72866	-0.07565
O	0.68386	0.73831	-0.0623
O	0.67595	0.74912	0.1147
H	1.17494	1.46128	-0.04494
H	1.0165	1.45997	-0.03788

H	0.80045	1.08397	0.08684
H	0.79948	1.24091	0.08248
H	1.18074	0.85466	-0.00679
H	1.02229	0.85482	-0.00225
H	1.41526	1.24341	0.0346
H	1.41703	1.08639	0.03686
H	0.82954	0.99203	-0.11564
H	0.712	0.89824	-0.11533
H	0.81194	0.80898	0.17092
H	0.92796	0.9043	0.17112
H	1.29354	0.91739	-0.14261
H	1.40004	0.8128	-0.14349
H	1.46906	0.87142	0.17309
H	1.36	0.97416	0.17415
H	1.27911	1.42709	0.15038
H	1.37554	1.54096	0.14688
H	1.45966	1.44772	-0.1396
H	1.36172	1.33561	-0.13661
H	0.93799	1.42946	0.15796
H	0.82889	1.53108	0.16003
H	0.70858	1.41962	-0.11232
H	0.81956	1.3188	-0.11485
Co	0.60568	0.6606	0.10145
Co	0.60489	0.65718	-0.06961
N	0.60568	0.65935	0.24791
C	0.59456	0.72919	0.30231
C	0.59428	0.72987	0.40834
C	0.6054	0.65816	0.45955
C	0.61667	0.58703	0.40208
C	0.61667	0.5889	0.2961
C	0.60525	0.65768	0.57215
C	0.59775	0.7294	0.62967
C	0.59781	0.72783	0.73561

N	0.60499	0.65708	0.78384
C	0.61231	0.58658	0.72944
C	0.61263	0.58562	0.62333

Supplementary Table 8. Cell space groups, lattice constants, and atomic coordinates of PPF-3.

Atoms	P1, *a* = 15.5485 Å, *b* = 15.5485 Å, *c* = 13.1532 Å, *α* = *β* = *γ* = 90°		
	x	*y*	*z*
C	1.04802	1.40749	-0.11631
N	1.11637	1.35516	-0.11651
C	1.18264	1.40828	-0.12969
C	1.15413	1.49651	-0.14096
C	1.07221	1.49597	-0.13293
C	0.96539	1.38333	-0.10057
C	0.94292	1.29894	-0.08618
N	0.99566	1.23064	-0.08945
C	0.94435	1.16253	-0.07669
C	0.8618	1.188	-0.06265
C	0.86095	1.27427	-0.06844
C	0.96864	1.07731	-0.07832
C	1.05253	1.05277	-0.08543
N	1.11967	1.106	-0.09442
C	1.18745	1.05307	-0.0984
C	1.16156	0.96363	-0.09055
C	1.07942	0.96343	-0.08365
C	1.2711	1.07773	-0.10852
C	1.2946	1.16311	-0.11688
N	1.24132	1.23123	-0.11795
C	1.29294	1.30009	-0.12695
C	1.37681	1.2755	-0.13123
C	1.3778	1.18889	-0.12572
C	1.2672	1.38506	-0.13163
C	0.90176	1.0108	-0.07418

C	1.33732	1.01009	-0.11031
C	0.8987	1.45002	-0.09953
C	1.33123	1.45448	-0.13826
C	0.84545	1.00124	-0.13987
C	0.77692	0.94558	-0.13365
C	0.76439	0.89714	-0.06237
C	0.82187	0.90567	0.00303
C	0.88967	0.96267	-0.00271
C	1.34537	0.95633	-0.17867
C	1.40703	0.89227	-0.18011
C	1.46231	0.88161	-0.11335
C	1.45422	0.93584	-0.04526
C	1.39179	0.99904	-0.04339
C	1.34221	1.51206	-0.07323
C	1.39695	1.58158	-0.08118
C	1.44331	1.59385	-0.15352
C	1.43279	1.53547	-0.21849
C	1.37668	1.46679	-0.21103
C	0.89483	1.50904	-0.03509
C	0.8313	1.57089	-0.03337
C	0.76996	1.57387	-0.0958
C	0.77345	1.51376	-0.1595
C	0.83789	1.45293	-0.16193
C	0.70032	1.63935	-0.09618
C	1.50364	1.66853	-0.15845
C	0.69003	0.83716	-0.05906
C	1.53323	0.81724	-0.11461
O	0.70888	1.69729	-0.03179
O	0.70066	1.68309	-0.17041
O	1.54877	1.66788	-0.23199
O	1.55926	1.66437	-0.09229
O	1.53115	0.76432	-0.04585
O	1.53684	0.76988	-0.1874

O	0.69432	0.78114	-0.12561
O	0.68488	0.79496	0.01665
H	1.19159	1.55255	-0.15472
H	1.03183	1.55145	-0.13875
H	0.80738	1.1485	-0.04932
H	0.80571	1.31421	-0.06076
H	1.20093	0.90709	-0.09053
H	1.04073	0.90681	-0.07736
H	1.43198	1.31574	-0.13786
H	1.43391	1.14943	-0.12772
H	0.85352	1.0387	-0.19511
H	0.73345	0.9402	-0.18464
H	0.81422	0.86931	0.05893
H	0.93208	0.9702	0.04885
H	1.30374	0.96407	-0.2308
H	1.41192	0.85211	-0.23388
H	1.49667	0.92942	0.00642
H	1.38634	1.03995	0.00987
H	1.30694	1.50397	-0.01693
H	1.40366	1.62571	-0.03048
H	1.46711	1.54292	-0.27535
H	1.36811	1.42355	-0.26215
H	0.94111	1.50706	0.01375
H	0.83024	1.61573	0.01708
H	0.72654	1.51448	-0.20782
H	0.83994	1.40739	-0.21185
Co	0.61373	0.70079	0.01368
Co	0.61275	0.69635	-0.12372
N	0.61381	0.69936	0.13176
C	0.60326	0.7739	0.1737
C	0.60304	0.77451	0.25915
C	0.6136	0.69787	0.30237
C	0.62429	0.62196	0.25798

C	0.62428	0.62407	0.17251
C	0.61344	0.69723	0.39313
C	0.60741	0.77377	0.4373
C	0.60736	0.77198	0.52272
N	0.61299	0.69635	0.56375
C	0.61889	0.62111	0.52208
C	0.61927	0.6202	0.43658
Co	2.13193	1.22645	-0.16596

Supplementary Table 9. Cell space groups, lattice constants, and atomic coordinates of HKUST-1.

Atoms	Fm-3m, $a = b = c = 26.3034$ Å, $\alpha = \beta = \gamma = 90°$		
	x	y	z
Cu	5.6847	20.6187	0
O	4.9406	19.5179	-1.2899
O	4.1244	22.179	0
C	5.3767	18.5284	-1.8391
C	4.7641	17.9158	-3.057
C	5.3333	16.7776	-3.6259
H	6.0763	16.4063	-3.2546
O	1.2899	18.0923	-6.3662
C	1.8391	18.5284	-5.3767
C	3.057	17.9158	-4.7641
C	3.6259	18.485	-3.6259
H	3.2546	19.228	-3.2546
O	4.9406	14.4416	-6.3662
C	5.3767	14.9908	-5.3767
C	4.7641	16.2087	-4.7641
O	6.3662	14.4416	-4.9406
C	3.6259	16.7776	-5.3333
H	3.2546	16.4063	-6.0763
O	6.3662	18.0923	-1.2899
O	1.2899	19.5179	-4.9406

Cu	0	20.6187	-5.6847
O	1.2899	21.3628	-6.7855
O	0	22.179	-4.1244
C	1.8391	20.9267	-7.775
C	3.057	21.5393	-8.3876
C	3.6259	20.9701	-9.5258
H	3.2546	20.2271	-9.8971
O	6.3662	25.0135	-8.2111
C	5.3767	24.4643	-7.775
C	4.7641	23.2464	-8.3876
C	3.6259	22.6775	-7.8184
H	3.2546	23.0488	-7.0754
O	6.3662	21.3628	-11.8618
C	5.3767	20.9267	-11.3126
C	4.7641	21.5393	-10.0947
O	4.9406	19.9372	-11.8618
C	5.3333	22.6775	-9.5258
H	6.0763	23.0488	-9.8971
O	1.2899	19.9372	-8.2111
O	4.9406	25.0135	-6.7855
Cu	5.6847	26.3034	-5.6847
O	4.1244	26.3034	-4.1244
Cu	5.6847	18.8364	-13.1517
Cu	0	18.8364	-7.467
Cu	5.6847	13.1517	-7.467
Cu	7.467	20.6187	-13.1517
Cu	7.467	26.3034	-7.467
Cu	7.467	18.8364	0
Cu	7.467	13.1517	-5.6847
O	6.7855	25.0135	-4.9406
O	6.7855	21.3628	1.2899
O	4.9406	27.5933	-6.7855
O	-1.2899	19.5179	-4.9406

O	4.9406	19.5179	1.2899
O	-1.2899	21.3628	-6.7855
O	6.7855	27.5933	-4.9406
O	6.7855	21.3628	-1.2899
C	4.1743	4.1743	4.1743
C	10.019	13.1517	10.019

Supplementary Table 10. Cell space groups, lattice constants, and atomic coordinates of ZIF-67.

Atoms	I-43m, $a = b = c = 16.9589$ Å, $\alpha = \beta = \gamma = 90°$		
	x	y	z
Co	0	8.4794	12.7192
N	1.5263	9.0357	13.8639
C	2.0809	8.3862	14.878
C	1.5907	7.0464	15.3682
C	2.2233	10.2177	13.7944
H	2.0605	10.9198	13.1754
Co	4.2397	8.4795	16.9589
Co	-4.2397	8.4795	16.9589
N	3.095	9.0357	15.4326
N	-1.5263	7.9232	13.8639
N	-3.095	7.9232	15.4326
N	0.5563	6.9531	11.5744
N	-0.5563	10.0058	11.5744
C	-2.0809	8.5727	14.878
C	-1.5907	9.9125	15.3682
C	3.1645	10.2177	14.7356
C	-2.2233	6.7412	13.7944
C	-3.1645	6.7412	14.7356
H	3.7835	10.9198	14.8984
H	-2.0605	6.0391	13.1754
H	-3.7835	6.0391	14.8984
H	2.3386	6.4139	15.3919

H	0.8988	6.7106	14.761
H	1.216	7.1465	16.267

Supplementary Table 11. Cell space groups, lattice constants, and atomic coordinates of ZIF-8.

Atoms	I-43M, $a = b = c = 16.99$ Å, $\alpha = \beta = \gamma = 90°$		
	x	*y*	*z*
Zn	0	4.2475	8.495
N	0.5182	5.4011	10.0207
C	-0.1529	6.3967	10.5933
C	1.733	5.3451	10.6986
H	2.4417	4.7391	10.5204
C	-1.507	6.8928	10.0972
N	0.5182	6.9693	11.5889
C	1.733	6.2914	11.6449
H	2.4417	6.4696	12.2509
N	-0.5182	5.4011	6.9693
C	0.1529	6.3967	6.3967
C	-1.733	5.3451	6.2914
H	-2.4417	4.7391	6.4696
C	1.507	6.8928	6.8928
N	-0.5182	6.9693	5.4011
C	-1.733	6.2914	5.3451
H	-2.4417	6.4696	4.7391
Zn	0	8.495	4.2475
Zn	0	8.495	12.7425
N	1.5257	3.0939	7.9768
N	-1.5257	3.0939	9.0132
H	-1.4249	7.8227	9.7992
H	-1.8051	6.335	9.3486
H	-2.1614	6.8395	10.8249

Supplementary Table 12. Detailed configuration parameters of the simulation models.

Number of molecules	Light	Solvothermal
Co-MOF	25	25
BPY	100	100
PVP	5	5
DMF	900	900
ethanol	300	300

References

1 Mastronarde, D. N. SerialEM: a program for automated tilt series acquisition on Tecnai microscopes using prediction of specimen position. *Microsc. Microanal.* **9**, 1182-1183 (2003).

2 Kremer, J. R., Mastronarde, D. N. & McIntosh, J. R. Computer visualization of three-dimensional image data using IMOD. *J. Struct. Biol.* **116**, 71-76 (1996).

3 Pettersen, E. F. *et al.* UCSF Chimera—a visualization system for exploratory research and analysis. *J. Comput. Chem.* **25**, 1605-1612 (2004).

4 Henry, E. R., Eaton, W. A. & Hochstrasser, R. M. Molecular dynamics simulations of cooling in laser-excited heme proteins. *Proc. Natl. Acad. Sci. U. S. A.* **83**, 8982-8986 (1986).

5 Greene, B., Hochstrasser, R., Weisman, R. & Eaton, W. Spectroscopic studies of oxy-and carbonmonoxyhemoglobin after pulsed optical excitation. *Proc. Natl. Acad. Sci. U. S. A.* **75**, 5255-5259 (1978).

6 Malde, A. K. *et al.* An automated force field topology builder (ATB) and repository: version 1.0. *J. Chem. Theory Comput.* **7**, 4026-4037 (2011).

7 Oostenbrink, C., Villa, A., Mark, A. E. & Van Gunsteren, W. F. A biomolecular force field based on the free enthalpy of hydration and solvation: The GROMOS force-field parameter sets 53A5 and 53A6. *J. Comput. Chem.* **25**, 1656-1676 (2004).

8 Yamashige, H., Matsuo, S., Kurisaki, T., Perera, R. C. C. & Wakita, H. Local

structure of nitrogen atoms in a porphine ring of meso-phenyl substituted porphyrin with an electron-withdrawing group using X-ray photoelectron spectroscopy and X-ray absorption spectroscopy. *Anal. Sci.* **21**, 635-639 (2005).

9 Eskelsen, J. R. *et al.* Protonation state of core nitrogens in the meso-tetra(4-carboxyphenyl)porphyrin impacts the chemical and physical properties of nanostructures formed in acid solutions. *J. Porphyrins Phthalocyanines* **16**, 1233-1243 (2012).
