# Supplementary material for: Room temperature photochemical synthesis of metal–organic frameworks for enhanced photocatalysis
Source: Nat Commun. 2026 Mar 20;17:4274. doi: 10.1038/s41467-026-70927-w (PMC13168299; doi:10.1038/s41467-026-70927-w)
Supplement: Supplementary file 2 — Descriptions of Additional Supplementary Files [file 41467_2026_70927_MOESM2_ESM.pdf]

Description of Additional Supplementary Files

File Name: Supplementary Data 1
Description: The XYZ file for the initial configuration of phoPPF-3.

File Name: Supplementary Data 2
Description: The XYZ file for the final configuration of phoPPF-3.

File Name: Supplementary Data 3
Description: The XYZ file for the initial configuration of PPF-3.

File Name: Supplementary Data 4
Description: The XYZ file for the final configuration of PPF-3.

File Name: Supplementary Data 5
Description: The XYZ file for the optimized TCPP structure.

File Name: Supplementary Data 6
Description: The XYZ file for the optimized TCPP* structure.
